# Retinal perivascular macrophages regulate immune cell infiltration during neuroinflammation in mouse models of ocular disease

**DOI:** 10.1172/JCI180904

**Published:** 2024-08-29

**Authors:** Jacob K. Sterling, Amrita Rajesh, Steven Droho, Joyce Gong, Andrew L. Wang, Andrew P. Voigt, C. Elysse Brookins, Jeremy A. Lavine

**Affiliations:** 1 Department of Medicine, Feinberg School of Medicine,; 2 Physician Scientist Track Program, Internal Medicine Residency, and; 3 Department of Ophthalmology, Feinberg School of Medicine, Northwestern University, Chicago, Illinois, USA.

**Keywords:** Ophthalmology, Macrophages, Monocytes, Retinopathy

## Abstract

The blood-retina barrier (BRB), which is disrupted in diabetic retinopathy (DR) and uveitis, is an important anatomical characteristic of the retina, regulating nutrient, waste, water, protein, and immune cell flux. The BRB is composed of endothelial cell tight junctions, pericytes, astrocyte end feet, a collagen basement membrane, and perivascular macrophages. Despite the importance of the BRB, retinal perivascular macrophage function remains unknown. We found that retinal perivascular macrophages resided on postcapillary venules in the superficial vascular plexus and expressed MHC class II. Using single-cell RNA-Seq, we found that perivascular macrophages expressed a prochemotactic transcriptome and identified platelet factor 4 (*Pf4*, also known as CXCL4) as a perivascular macrophage marker. We used *Pf4^Cre^* mice to specifically deplete perivascular macrophages. To model retinal inflammation, we performed intraocular CCL2 injections. Ly6C^+^ monocytes crossed the BRB proximal to perivascular macrophages. Depletion of perivascular macrophages severely hampered Ly6C^+^ monocyte infiltration. These data suggest that retinal perivascular macrophages orchestrate immune cell migration across the BRB, with implications for inflammatory ocular diseases including DR and uveitis.

## Abstract 2

Perivascular macrophages are a component of the blood-retina barrier with unknown function. Perivascular macrophages express chemokines and regulate immune cell infiltration into the retina.

## Graphical abstract

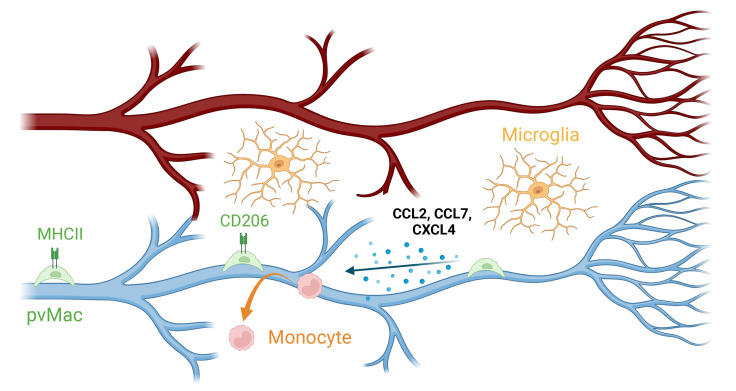

## Introduction

The blood vessels of the CNS possess unique properties that allow them to regulate the flux of molecules and cells between the systemic circulation and the tightly controlled environment of the CNS. The blood-retina barrier (BRB), which is analogous to the blood-brain barrier, is an important anatomical characteristic of the retina, regulating nutrient, waste, water, protein, and immune cell flux. Disruption of the BRB leads to vasogenic edema, immune cell infiltration, and neurodegeneration across multiple ocular diseases. In diabetic retinopathy (DR), for example, the BRB disruption leads to leaky vasculature and correlates with DR severity (1). In retina-involved noninfectious and infectious uveitis, BRB dysfunction is exemplified by cystoid macular edema with petaloid leakage, fern-like retinal vasculitis in intermediate uveitis, Kyrieleis plaques in toxoplasmosis, and potentially gass plaques in Susac syndrome (2).

At the cellular and molecular levels, the BRB is composed of endothelial cell tight junctions, pericytes, astrocyte end feet, a collagen IV basement membrane, and perivascular macrophages (3). Although many studies have interrogated the function of the BRB and its individual components, retinal perivascular macrophages have been underinvestigated, and their function remains largely unknown. Perivascular macrophages were first noted in the literature in the 1950s (4). Subsequent work to identify the function of these cells has been limited. In 2009, one manuscript demonstrated that IBA1^neg^F4/80^+^CD11b^+^ macrophages, which were described as perivascular macrophages, migrate along blood vessels to sites of BRB breakdown (5). Recently, select publications have noted changes in possible perivascular macrophage morphology and number in mouse models of DR (6), retinal vein occlusion (7), and light damage (8). Of note, the markers used in these studies to identify perivascular macrophages included CD14, F4/80, and IBA1, which are inadequate to differentiate perivascular macrophages from infiltrating monocytes and monocyte-derived macrophages.

Compared with retinal perivascular macrophages, brain perivascular macrophages have been studied in greater detail. Recent landmark publications have shown that brain perivascular macrophages are derived from meningeal macrophages, which are derived from the yolk sac and fetal liver, without contribution from the bone marrow. In the adult mouse, brain perivascular macrophages occupy a unique niche within the collagen IV basement membrane of both venules and arterioles, but not capillaries, forming a critical piece of the blood-brain barrier (9). The potential functions of brain perivascular macrophages include maintenance of the blood-brain barrier and mediation of neurovascular and cognitive dysfunction during both hypertension and Alzheimer’s disease, which are associated with blood-brain barrier hyperpermeability (3, 10).

Previous work has shown that outside the CNS, blood vessel–associated macrophages mediate immune cell recruitment via crosstalk with endothelial cells (11). In addition, immune cell recruitment occurs at postcapillary venules and not capillaries (12). Given the physical proximity of retinal perivascular macrophages and retinal endothelial cells, and the aforementioned links between BRB dysfunction and peripheral immune cell recruitment, we hypothesized that retinal perivascular macrophages regulate immune cell infiltration during neuroinflammation.

We previously showed that retinal perivascular macrophages are identifiable in the mouse retina as Tmem119^neg^CD206^+^IBA1^+^ elongated cells that are adjacent to CD31^+^ vessels and within the collagen IV perivascular sheath (13). In this study, we investigated the anatomical locations of retinal perivascular macrophages, identified a mouse model to target them, used prior single-cell RNA-Seq (scRNA-Seq) data to infer perivascular macrophage function, and tested whether their ablation affected neuroinflammation. We found that retinal perivascular macrophages resided on postcapillary venules, could be targeted with platelet factor 4–Cre (*Pf4^Cre^*) mice, and expressed a prochemotactic transcriptional profile, and that their ablation decreased immune cell infiltration during acute neuroinflammation. Together, these data point to perivascular macrophages as key mediators of retinal inflammation, a pathogenic feature of multiple retinal inflammatory and degenerative diseases.

## Results

**Figure 1:**
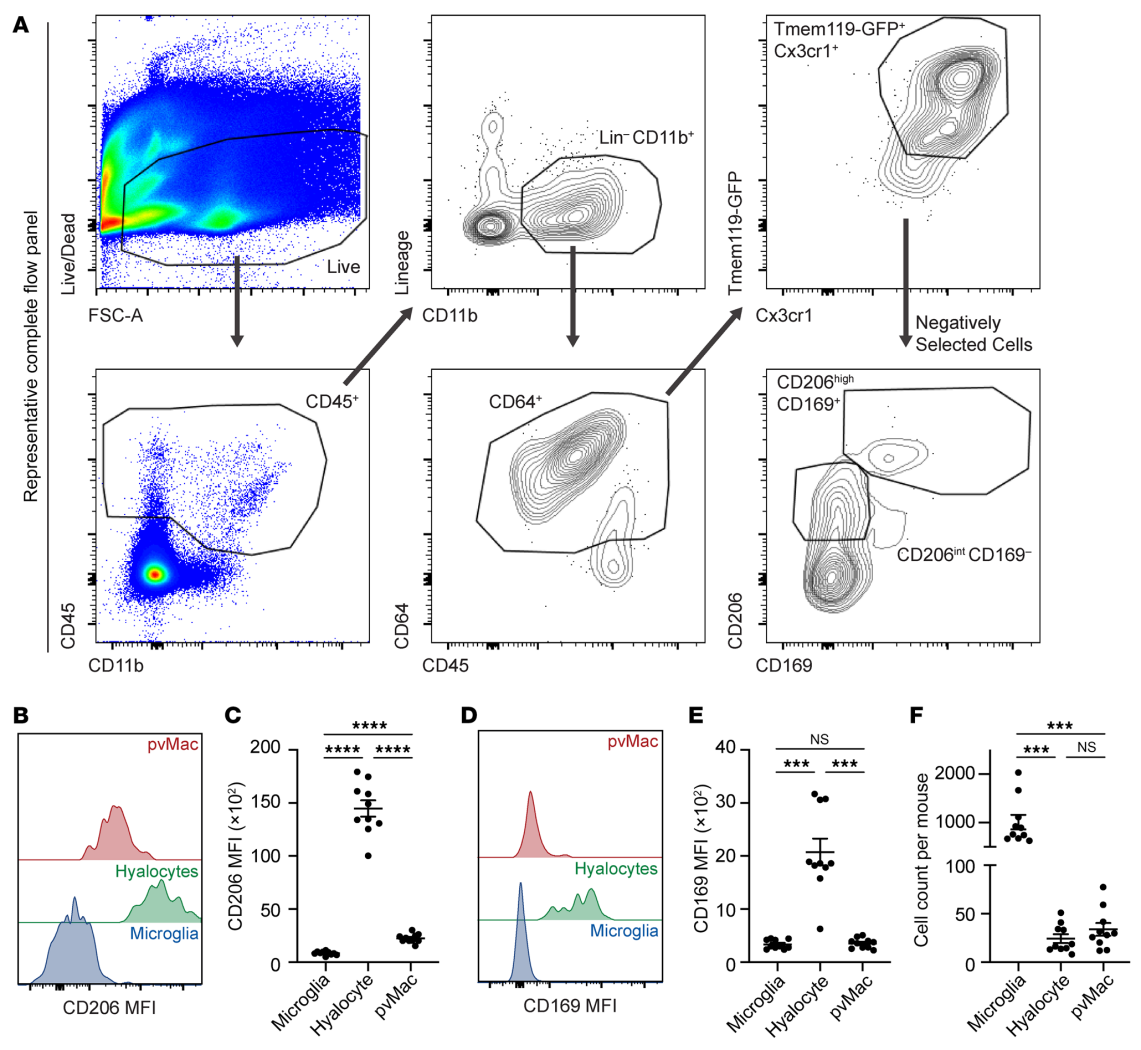
Flow cytometric identification of retinal macrophage heterogeneity. (**A**) Flow cytometric gating strategy. Top left panel: Live cells were identified from singlets. Bottom left panel: CD45^+^ cells were identified from live, singlet cells. Top middle panel: Lineage (CD4^+^, CD8^+^ [T cells], B220 [B cells], Ly6G [neutrophils], NK1.1 [NK cells], SiglecF [eosinophils]) versus CD11b plot to gate forward CD11b^+^Lin^neg^ mononuclear phagocytes. Bottom middle panel: CD64^+^ macrophages were identified. Top right panel: Tmem119^GFP^ versus Cx3cr1 plot to delineate Cx3cr1^hi^Tmem119^GFP+^ microglia. Bottom right panel: Nonmicroglia were plotted on a CD206 versus CD169 plot to identify CD206^hi^CD169^+^ hyalocytes and CD206^int^CD169^neg^ perivascular macrophages. FSC-A, forward scatter area. (**B**) Modal frequency histogram for CD206 expression. (**C**) Hyalocytes expressed the most CD206, while perivascular macrophages expressed intermediate CD206 levels. (**D**) Modal frequency histogram for CD169 expression. (**E**) Hyalocytes expressed the most CD169, whereas perivascular macrophages expressed low levels of CD169. (**F**) Microglia were the most abundant retinal macrophages, followed by perivascular macrophages and hyalocytes. Data are presented as the mean ± SEM. ***P < 0.001 and ****P < 0.0001, by repeated-measures, 1-way ANOVA followed by Tukey’s multiple-comparison test. *n* = 10 per group. pvMac, perivascular macrophages.

For a comprehensive characterization of perivascular macrophages, we performed multiparameter flow cytometry of retinal tissue from *Tmem119^GFP/+^* mice, which label microglia as GFP^+^ with high sensitivity in the brain and retina (13, 14). After singlet gating, live CD45^+^ cells were gated forward (Figure 1A, left). Next, CD11b^+^lineage^neg^ (lineage = B cells, T cells, NK cells, eosinophils, and neutrophils) cells were isolated (Figure 1A, top middle). CD64 was used to identify all macrophages (Figure 1A, bottom middle). Microglia were defined as Cx3cr1^hi^GFP^+^ cells (Figure 1A, top right). We used CD169 to discriminate vitreal hyalocytes on the surface of the retina from perivascular macrophages (13, 15). We defined perivascular macrophages as CD206^dim^CD169^neg^ and hyalocytes as CD206^+^CD169^+^ from nonmicroglial macrophages (Figure 1A, bottom right). The fluorescence minus one (FMO) control for CD169 is shown in Supplemental Figure 1 (supplemental material available online with this article; https://doi.org/10.1172/JCI180904DS1). Quantitative analysis confirmed that perivascular macrophages were CD206^dim^ (Figure 1, B and C) and expressed very low levels of CD169 compared with hyalocytes (Figure 1, D and E). Quantitative analysis of cell counts per mouse revealed that perivascular macrophages were the second most abundant macrophage subtype in the retina (Figure 1F).

**Figure 2:**
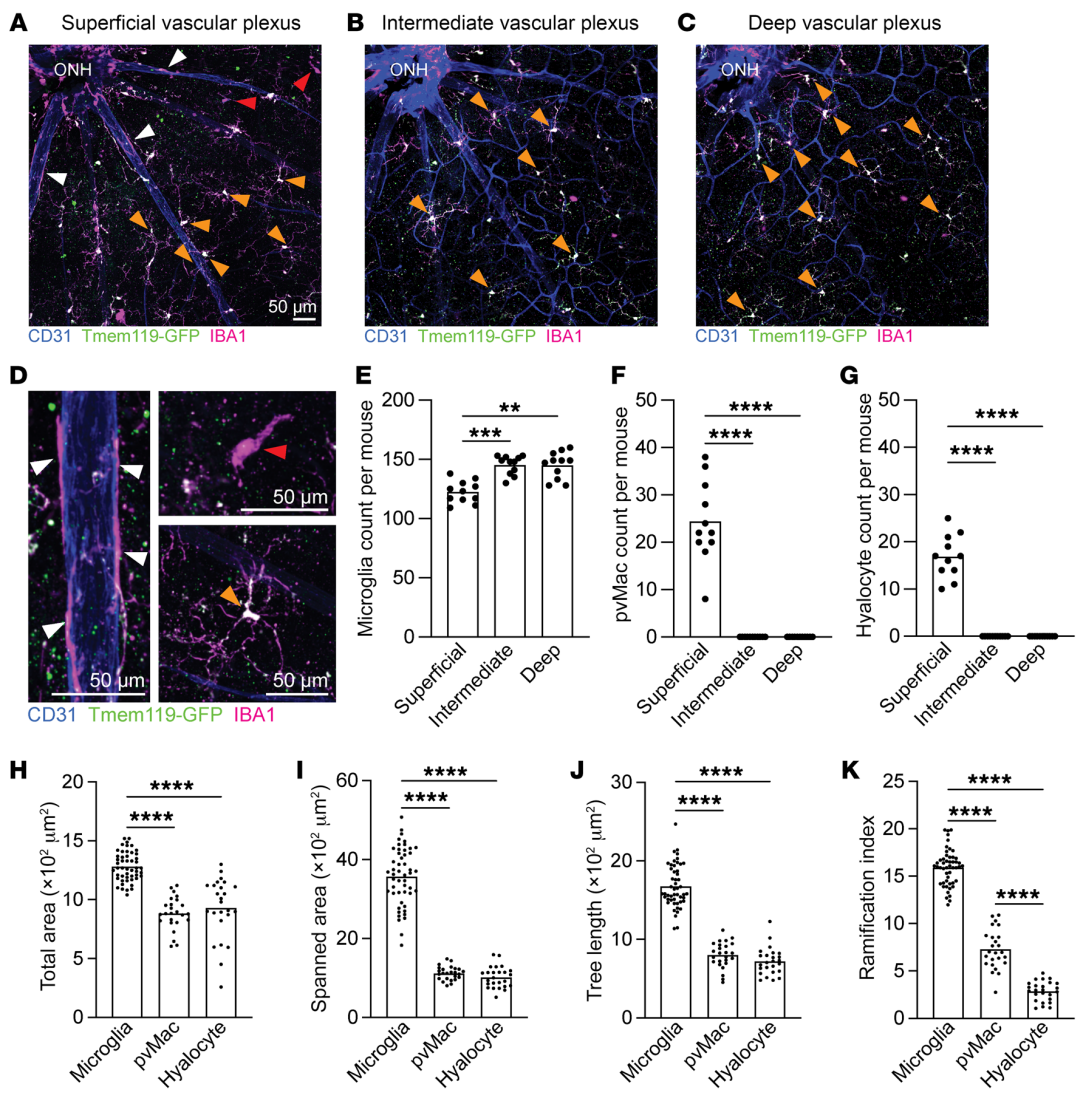
Perivascular macrophages only reside in the superficial vascular plexus. (**A**–**C**) In the representative images of superficial (**A**), intermediate (**B**), and deep (**C**) vascular plexuses, white arrowheads identify perivascular macrophages, orange arrowheads show microglia, and red arrowheads highlight hyalocytes. Scale bar: 50 μm. (**D**) Zoomed-in representative image of each cell type. Scale bars: 50 μm. (**E**–**G**) Counts of microglia (**E**), periventricular macrophages (**F**), and hyalocytes (**G**) in each vascular plexus. Microglia were in all 3 plexuses, while perivascular macrophages and hyalocytes were only found in the superficial vascular plexus. **P < 0.01, ***P < 0.001, and ****P < 0.0001, by repeated-measures, 1-way ANOVA followed by Tukey’s multiple-comparison test. (**H**–**K**) MotiQ quantitative morphometry showing total area, spanned area, tree length, and ramification index. ****P < 0.0001, by 1-way ANOVA followed by Tukey’s multiple-comparison test. *n* = 11 per group (**E**–**G**); *n* = 25–50 cells per group (**H**–**K**). Data are presented as the mean ± SEM. ONH, optic nerve head.

Next, we performed confocal microscopy of retinal flatmounts from *Tmem119^GFP/+^* mice to determine where perivascular macrophages reside anatomically. Retinas were stained for CD31 to visualize vasculature and IBA1 to identify all macrophages. Microglia were defined as IBA1^+^GFP^+^ ramified cells (Figure 2, A–D, orange arrowheads). Perivascular macrophages were identified as IBA1^+^GFP^neg^ cells along CD31^+^ vessels (white arrowheads). Hyalocytes were delineated as IBA1^+^GFP^neg^ nonramified cells not on vessels (red arrowheads). Microglia were found in all vascular plexuses (Figure 2E), while perivascular macrophages and hyalocytes were only found in the superficial vascular plexus (Figure 2, F and G). Next, we performed MotiQ morphometric analysis (16) on microglia (*n* = 50 cells), perivascular macrophages (*n* = 25 cells), and hyalocytes (*n* = 25 cells) from 5–6 mice per group. We found that microglia had a greater total area, spanned area (polygonal area connecting the outer points of the dendritic arbor), and tree length (sum of dendrites) compared with both perivascular macrophages and hyalocytes (Figure 2, H–J). Additionally, hyalocytes were the least ramified, whereas perivascular macrophages were intermediate and microglia had the highest ramification index (Figure 2K).

**Figure 3:**
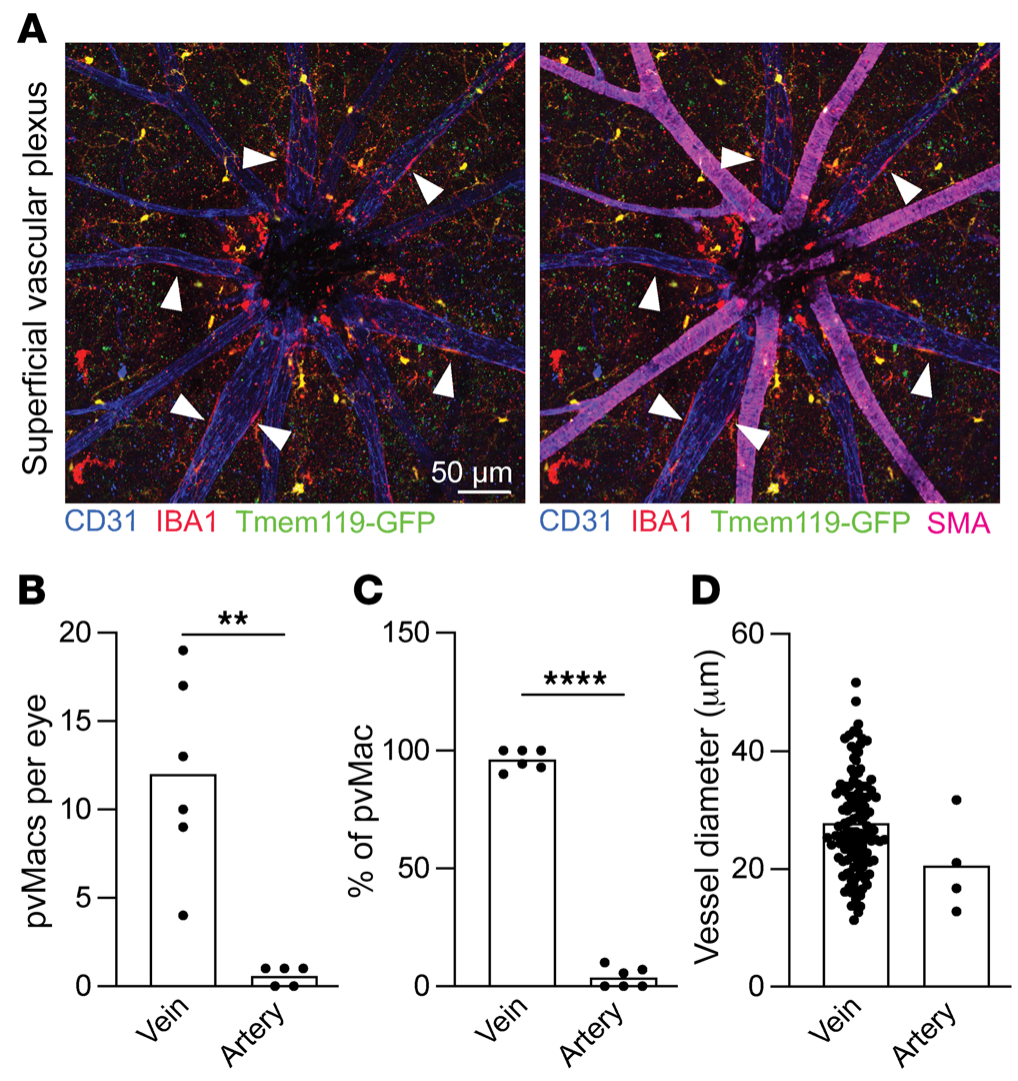
Perivascular macrophages reside on venules. (**A**) Representative images of the superficial vascular plexus. White arrowheads identify perivascular macrophages on SMA^neg^ venules. Scale bar: 50 μm. (**B** and **C**) Number and percentage of perivascular macrophages on venules versus arterioles. (**D**) Perivascular macrophages reside on vessels with an average diameter of 25–30 μm. Data are presented as the mean ± SEM. **P < 0.01 and ****P < 0.0001, by 2-tailed *t* test. *n* = 5–6 mice per group (**B** and **C**). *n* = 129 vein and *n* = 4 artery perivascular macrophages (**D**).

Since the superficial vascular plexus contains arterioles and venules, while the intermediate and deep capillary plexuses are entirely capillaries, we next investigated whether perivascular macrophages reside on arterioles or venules. Smooth muscle actin (SMA) was used to identify arterioles (Figure 3A). Perivascular macrophages were found almost entirely on venules (Figure 3, B and C). We next measured the diameter of vessels where perivascular macrophages dwell. We found that perivascular macrophages reside on postcapillary venules with an average diameter of 27.8 μm (range, 11–51 μm, Figure 3D). Since postcapillary venules are the site of transendothelial migration of immune cells (12) and macrophage–endothelial cell crosstalk mediates immune cell tissue infiltration (11), we hypothesized that retinal perivascular macrophages may regulate immune cell infiltration into the retina.

**Figure 4:**
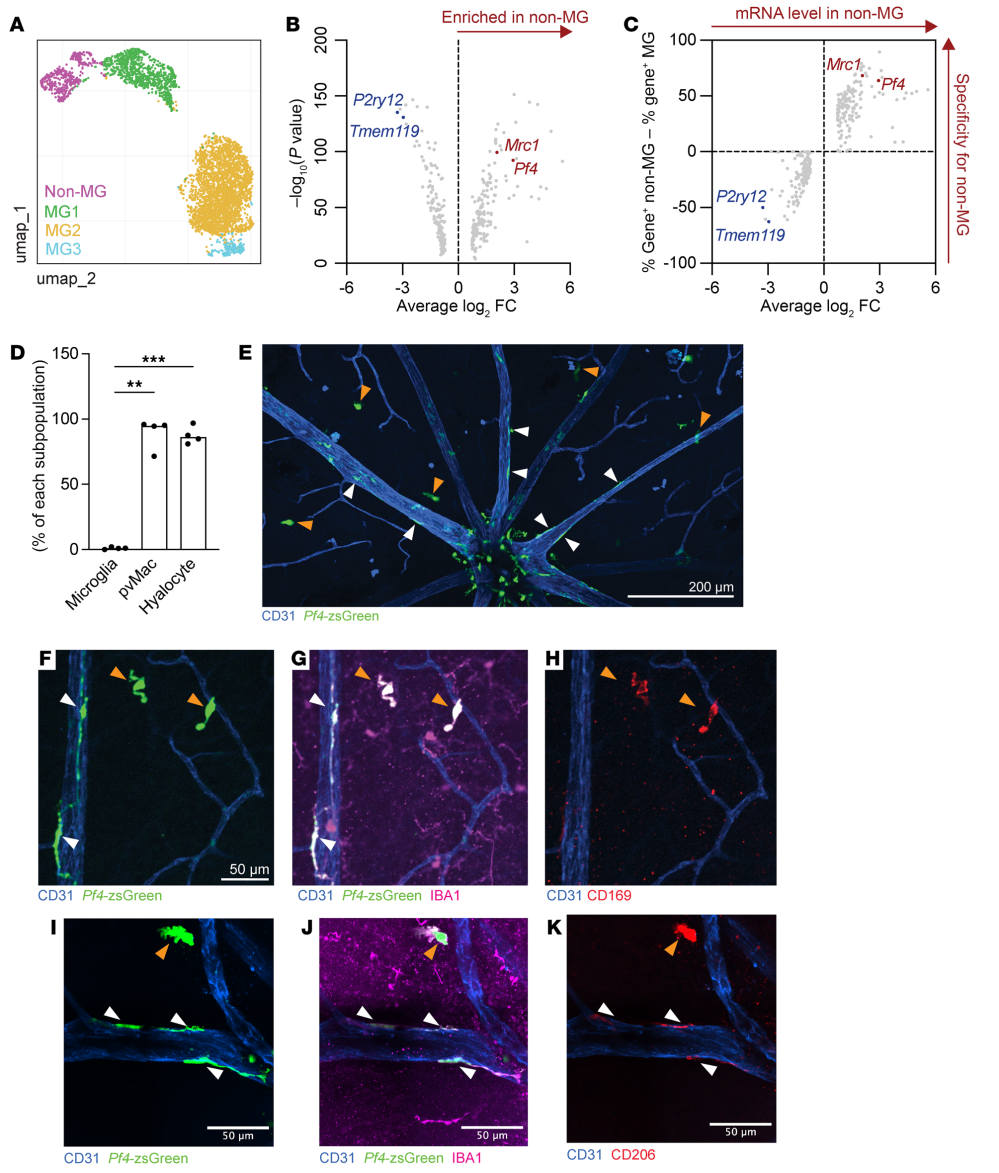
Perivascular macrophages express *Pf4*. (**A**) Uniform manifold approximation and projection (UMAP) dimension plot. (**B** and **C**) Microglia (MG) express higher levels of *P2ry12* and *Tmem119* than do non-MG. *Mrc1* and *Pf4* are more highly expressed in non-MG retinal macrophages. (**D**) Perivascular macrophages and hyalocytes were targeted by *Pf4^Cre^* to a significantly greater degree than microglia. **P < 0.01 and ***P < 0.001, by repeated-measures, 1-way ANOVA followed by Tukey’s multiple-comparison test. (**E**) Representative image of the superficial vascular plexus. Orange arrowheads delineate CD169^+^ hyalocytes; white arrowheads identify perivascular macrophages. Scale bar: 200 μm. (**F**–**H**) Representative zoomed-in images of *Pf4*-zsGreen perivascular macrophages and hyalocytes stained for CD169 (**F**–**H**) and CD206 (**I**–**K**). *n* = 4 per group for **D**. Images in **F**–**K** are representative of 3 mice. Scale bars: 50 μm. Data are presented as the mean ± SEM.

To test this hypothesis, we sought to create a genetic mouse model to target perivascular macrophages. We used previously published retinal scRNA-Seq data available on Spectacle (17, 18). We first identified 4 clusters of *Aif1*/IBA1^+^ tissue-resident macrophages present in WT mouse retinas (Figure 4A and Supplemental Figure 2, A and B). We hypothesized that these clusters would contain microglia, hyalocytes, and perivascular macrophages. Three of 4 clusters were strongly positive for the microglial markers *Tmem119* and *P2ry12* and were thus named microglia 1, 2, and 3 (MG1, MG2, MG3) (Supplemental Figure 2, C and D). The fourth cluster, named nonmicroglia (non-MG), did not express *Tmem119* or *P2ry12* (Supplemental Figure 2, C and D) but did express *Mrc1* (also known as CD206) (Supplemental Figure 2E). On the basis of this fourth cluster’s expression of *Mrc1*/CD206 (a marker of nonmicroglial tissue–resident macrophages such as perivascular macrophages, Figure 1) and lack of the microglial markers *Tmem119* and *P2ry12*, we hypothesized that the non-MG cluster was enriched with perivascular macrophages. Differential gene expression analysis comparing MG1, MG2, and MG3 in aggregate versus non-MG identified *Pf4* (also known as CXCL4) as a robustly expressed candidate marker of the non-MG cluster and perivascular macrophages (Figure 4, B and C).

To test the specificity of *PF4*/CXCL4 for perivascular macrophages, we bred *Pf4^Cre^* with *Rosa26^CAG-LSL-ZsGreen1^* reporter mice (hereafter referred to as *Pf4*-zsGreen mice). We found that perivascular macrophages (white arrowheads) and vitreal hyalocytes (orange arrowheads) were 89% and 88% zsGreen^+^, respectively, compared with microglia, which expressed zsGreen in 1% of cells (Figure 4, D and E). We performed staining for CD169 to confirm that zsGreen^+^ cells away from vessels were vitreal hyalocytes and not microglia (Figure 4, F–H). Additionally, we confirmed that both zsGreen^+^ perivascular macrophages and zsGreen^+^ hyalocytes were costained with CD206 (Figure 4, I–K), as expected from Figure 4, B and C. These data demonstrate that *Pf4^Cre^* mice had effective targeting of perivascular macrophages and hyalocytes.

The above analysis did not quantify the many zsGreen^+^ cells observed on the optic nerve head (Figure 4E). To further investigate these cells, we performed immunofluorescence of frozen sections through optic nerve heads from *Pf4*-zsGreen mice. Frozen sections were stained for neurofilament to determine whether these optic nerve head zsGreen^+^ cells were optic nerve head microglia. zsGreen^+^IBA1^+^CD31^neg^ cells (red arrowhead) on the vitreous side of the optic nerve head were identified as vitreal hyalocytes (Supplemental Figure 3, A–C). zsGreen^+^IBA1^+^CD31^+^ perivascular macrophages (white arrowhead) were also found at the optic nerve head (Supplemental Figure 3, A–C). zsGreen^neg^IBA1^+^neurofilament^+^ microglia (orange arrowhead) were detectable in the retina adjacent to the optic nerve head and in the optic nerve substance, but we found none at the optic nerve head (Supplemental Figure 3, A–D). These data suggest that optic nerve head zsGreen^+^ macrophages are a mixture of perivascular macrophages and vitreal hyalocytes.

To determine if brain perivascular macrophages also prefer venules to arterioles, we performed immunofluorescence imaging of frozen sections of brain from *Pf4*-zsGreen mice (representative images are shown in Supplemental Figure 4, A–C). Perivascular macrophages were identified as CD206^+^IBA1^+^CD31^adjacent^. Arterioles were discriminated from venules using SMA. Border-associated macrophages were delineated as CD206^+^IBA1^+^CD31^neg^ at the dural border of the brain. Microglia were defined as CD206^neg^IBA1^+^. In the brain, perivascular macrophages were 93% zsGreen^+^, while microglia were only 7.2% zsGreen^+^ (Supplemental Figure 4D), similar to retina. Unlike the retina, brain perivascular macrophages showed no preference for venules compared with arterioles (Supplemental Figure 4E).

**Figure 5:**
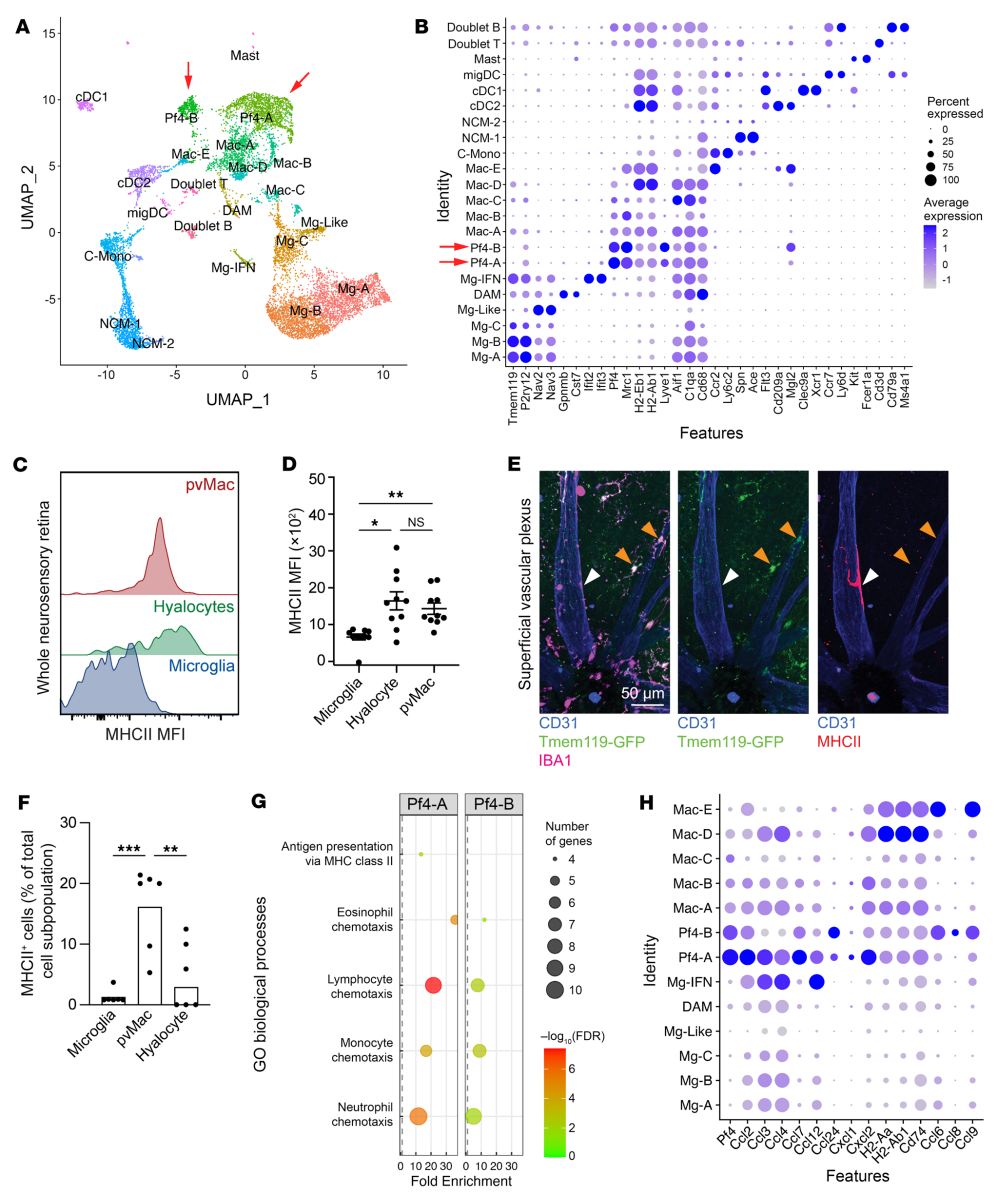
*Pf4^+^* cells are MHCII^+^ and prochemotactic. (**A**) UMAP dimension plot. (**B**) DotPlot of cluster-identifying genes showing 2 *Pf4*^+^ clusters (red arrows): Pf4-A and Pf4-B. (**C**) Modal frequency histogram of MHCII MFI from multiparameter flow cytometry. (**D**) Hyalocytes and perivascular macrophages expressed significantly higher levels of MHCII than did microglia, in agreement with the scRNA-Seq data. (**E**) Representative images of MHCII immunofluorescence in the superficial vascular plexus. White arrowheads identify MHCII^+^ perivascular macrophages, and orange arrowheads show MHCII^–^ hyalocytes. Scale bar: 50 μm. (**F**) Perivascular macrophages expressed significantly higher levels of MHCII than did microglia and hyalocytes. (**G**) GO enrichment for Pf4-A and Pf4-B showing increased expression of chemotaxis GO terms. (**H**) DotPlot of pro-chemotaxis genes differentially expressed in Pf4-A or Pf4-B. Size of dot indicates percentage of cells expressing the gene. Darkness of dot shows average expression with blue > purple. Data are presented as the mean ± SEM. *P < 0.05, **P < 0.01, and ***P < 0.001, by repeated-measures, 1-way ANOVA followed by Tukey’s multiple-comparison test (**D** and **F**). *n* = 6 per group.

To infer perivascular macrophage function, we reanalyzed recently published scRNA-Seq data from our laboratory (19, 20). This dataset included fluorescence-activated cell–sorted CD45^+^ cells from WT mouse eyes, *Ccr2^–/–^* mouse eyes, WT mouse retina/choroid, and *Nr4a1^–/–^* mouse retina/choroid. Of note, no laser-treated samples from the original study were included in the reanalysis. From this integrated, reclustered mononuclear phagocyte subset, we identified 3 *Tmem119^+^P2ry12^+^* microglia (Mg-A, Mg-B, and Mg-C) clusters, *Gpnmb^+^Cst7^+^* disease–associated microglia (DAM), *Ifit2^+^Ifit3^+^* IFN microglia (Mg-IFN), 2 *Pf4^+^Mrc1^+^* potential perivascular macrophage populations (Pf4-A and Pf4-B, red arrows), 5 *Aif1^+^C1qa^+^CD68^+^* macrophage clusters (Mac-A, Mac-B, Mac-C, Mac-D, and Mac-E), *Ly6c2^+^Ccr2^+^* classical monocytes (C-Mono), 2 *Ace^+^Spn^+^* nonclassical monocyte subsets (NCM-1 and NCM-2), 3 *Flt3*^+^ DC populations (cDC1, cDC1, and migDC), and *Fcer1a^+^Kit^+^* mast cells (Figure 5, A and B). The DAM population corresponded to previously published subretinal microglia (Supplemental Figure 5, A and B), which can be present at low numbers (~1%–3%, Supplemental Figure 5C) in young WT mice (17, 21, 22). Compared with microglia, we noted that Pf4-A and Pf4-B cells expressed moderate levels of the MHC class II (MHCII) genes *H2-Ab1* and *H2-Eb1* (Figure 5B). Using the multiparameter flow cytometric gating shown in Figure 1A, we found that both perivascular macrophages and hyalocytes expressed significantly higher levels of MHCII than did microglia (Figure 5, C and D). Additionally, we stained *Tmem119^GFP/+^* retina flatmounts for MHCII and found that perivascular macrophages were the predominant MHCII-expressing cells in the retina (Figure 5, E and F). On the basis of these data, we suspected that either Pf4-A or Pf4-B cells were perivascular macrophages. We next performed differential expression analysis (Supplemental Table 1) followed by gene ontology (GO) enrichment analysis (Supplemental Table 2). We found that Pf4-A was enriched for antigen presentation via MHCII (13.41-fold, *q* < 0.01), and chemotaxis of eosinophils (35.92-fold; genes = *Ccl2*, *Ccl4*, *Ccl7*, *Ccl12*, *Ccl24*; *q* < 0.001), lymphocytes (21.55-fold; genes = *Ccl2*, *Ccl3*, *Ccl4*, *Ccl7*, *Ccl12*, *Ccl24, Gas6, Gpr143, Ch25h*; *q* < 0.001), monocytes (16.76-fold; genes = *Ccl2*, *Ccl3*, *Ccl4*, *Ccl7*, *Ccl12*, *Ccl24;* *q* < 0.001), and neutrophils (11.70-fold; genes = *Cxcl1*, *Cxcl2*, C5ar1, *Ccl2*, *Ccl3*, *Ccl4*, *Ccl7*, *Ccl12*, *Ccl24, Pf4;* *q* < 0.001) (Figure 5, G and H). Similarly, Pf4-B demonstrated enrichment for chemotaxis of eosinophils (12.11-fold; genes = *Ccl2*, *Ccl7*, *Ccl8*, *Ccl24*; *q* < 0.05), lymphocytes (7.71-fold; genes = *Ccl2*, *Ccl6*, *Ccl7*, *Ccl8*, *Ccl9*, *Ccl24, Gas6;* *q* < 0.01), monocytes (8.93-fold, *q* < 0.01), and neutrophils (4.96-fold; genes = *Ccl2*, *Ccl6*, *Ccl7*, *Ccl8*, *Ccl9*, *Ccl24*, *Pf4*, *Pde4d*, *Vav3;* *q* < 0.01) (Figure 5, G and H). Based on these data, Pf4^+^ macrophages are enriched for chemotaxis genes, supporting our hypothesis that retinal perivascular macrophages may regulate immune cell infiltration into the retina.

**Figure 6:**
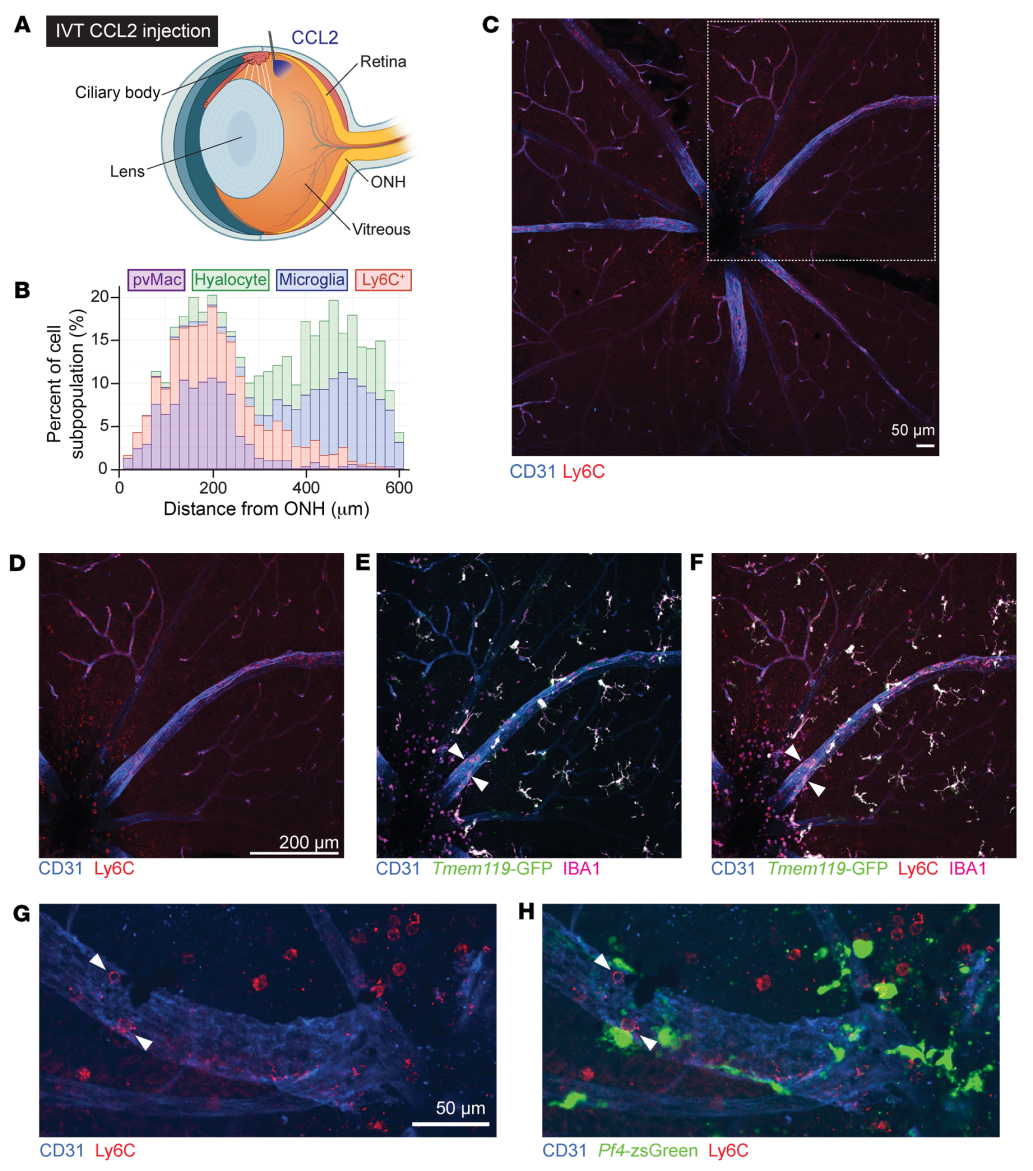
Perivascular macrophages correlate with Ly6C^+^ inflammatory cell infiltration. (**A**) Schematic showing approximate location for intravitreal injections of CCL2. (**B**) Frequency histogram of each cell type and their distance from the optic nerve. Significant overlap was found between the distribution of perivascular macrophages and Ly6C^+^ cells using a transformation of the Kolmogorov-Smirnov test. *n* = 2,998 cells. (**C**) Overview representative image of Ly6C^+^ cell infiltration at the superficial vascular plexus. Scale bar: 50 μm. (**D**–**F**) Zoomed-in representative images identifying Ly6C^+^ cells close to the optic nerve head where most perivascular macrophages (white arrowheads) resided in Tmem119^GFP^ mice. Scale bar: 200 μm. (**G** and **H**) Zoomed-in representative images of cells from *Pf4*-zsGreen mice showing close association between Ly6C^+^ monocytes (red cell, white arrowhead) and *Pf4^+^* perivascular macrophages. Scale bar: 50 μm.

To initially test this hypothesis, we injected the chemokine CCL2, which is increased during DR (23), into the vitreous of *Tmem119^GFP/+^* mice (Figure 6A). CCL2 binds the CCR2 receptor and recruits classical Ly6C^+^ monocytes to tissue. To our surprise, despite injecting CCL2 into the peripheral vitreous, the majority of infiltrating Ly6C^+^ cells were detected near the optic nerve (Figure 6, B–F). We quantitated the distance of each microglial cell, hyalocyte, perivascular macrophages, and Ly6C^+^ cell from the optic nerve. We found a significant correlation between the distributions of Ly6C^+^ cells and perivascular macrophages (*P* < 0.05). We repeated the CCL2 study in *Pf4*-zsGreen mice and found that intravascular Ly6C^+^ cells were detectable near zsGreen^+^ perivascular macrophages (Figure 6, G and H). Importantly, Ly6C^+^ cells did not express zsGreen, suggesting that classical monocytes and bone marrow precursors do not express *Pf4* immediately after tissue infiltration.

**Figure 7:**
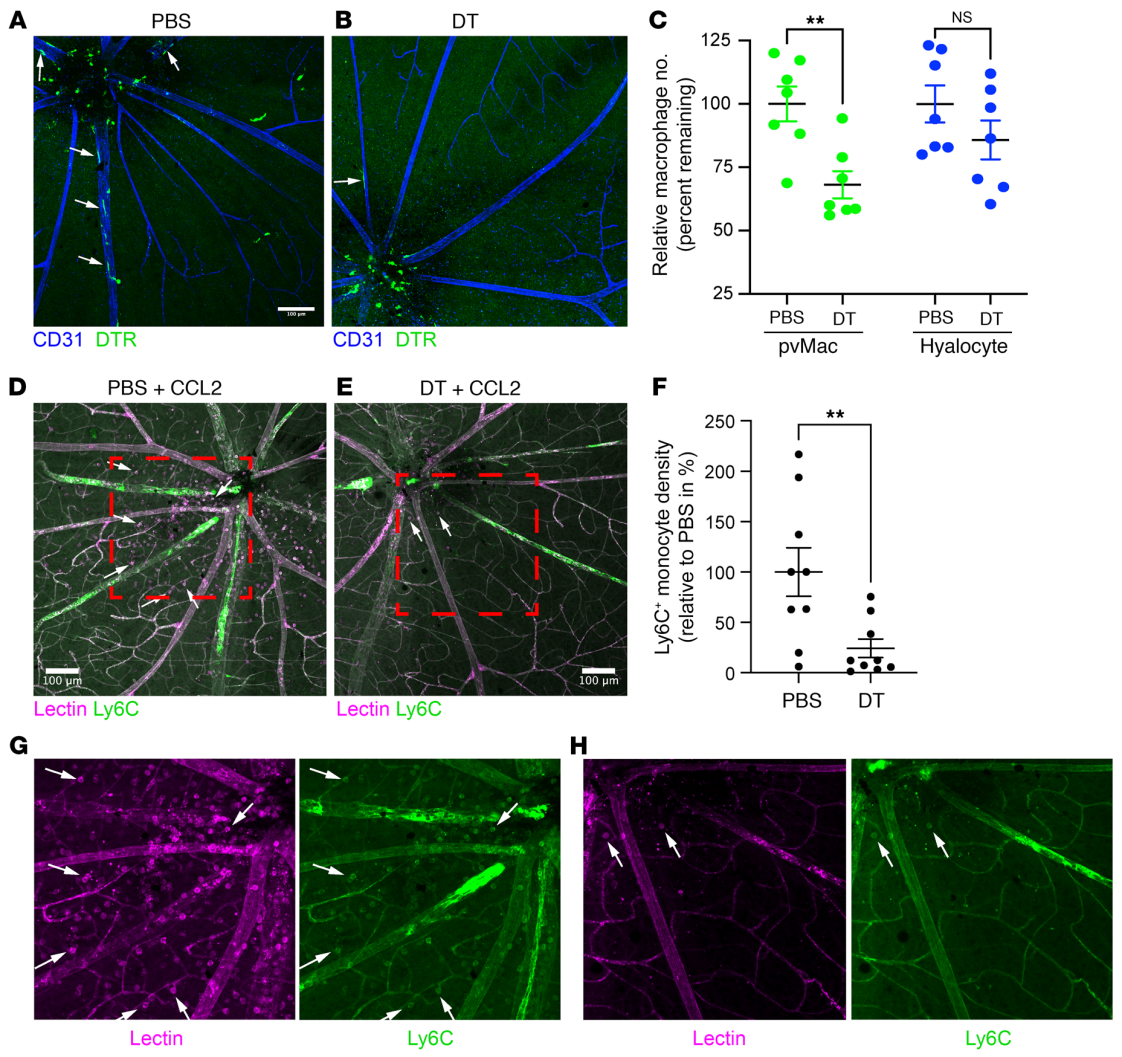
*Pf4-DTR* mice deplete perivascular macrophages and block immune cell infiltration. (**A** and **B**) Representative immunofluorescence images of the superficial vascular plexus from PBS- or DT-treated *Pf4-DTR* mice. White arrows identify DTR^+^ perivascular macrophages. Scale bar: 100 μm. (**C**) DT treatment reduced the number of perivascular macrophages without affecting hyalocytes. **P < 0.01, by 2-way ANOVA followed by Šidák’s multiple-comparison test. *n* = 7 per group. (**D** and **E**) Representative immunofluorescence images of the superficial vascular plexus from PBS- and DT-treated *Pf4^Cre^*:*R:26^CAG-LSL-DTR^* mice after intravitreal CCL2 injection. White arrows identify Ly6C^+^ infiltrating immune cells. Red dash-outlined boxes indicate enlarged areas in **G** and **H**. Scale bars: 100 μm. (**F**) DT treatment decreased the density of Ly6C^+^ monocytes after CCL2 injection. **P < 0.01, by Mann-Whitney *U* test because the distribution was nonparametric on the Shapiro-Wilk test. *n* = 9 per group. (**G** and **H**) Enlarged images from **D** and **E** separated by lectin and Ly6C channels for PBS (**G**) and DT (**H**) treatment. Data are presented as the mean ± SEM.

Next, we generated *Pf4^Cre^*:*Rosa26^CAG-LSL-DTR/+^* (*Pf4-DTR*) mice to deplete perivascular macrophages with diphtheria toxin (DT) injections. Ten- to 12-week-old *Pf4-DTR* mice were treated with vehicle PBS or DT for 4 days. Importantly, 4 days of DT treatment had no effect on animal health. On day 5, retinal flatmounts were stained for CD31, IBA1, and the DT receptor (DTR). We found that DT-treated mice had a 32% reduction in DTR^+^ retinal perivascular macrophages (*P* < 0.01) with no change in DTR^+^ hyalocytes (Figure 7, A–C) or retinal microglia (Supplemental Figure 6). The discrepancy between 32% perivascular macrophage ablation in *Pf4-DTR* mice compared with 89% targeting with *Pf4*-zsGreen (Figure 4F) is likely due to the low-dose DT (200 ng) or the fact that the same promoter can have differential efficiency even between Rosa26 reporter lines (24).

Next, we repeated the above study and performed fluorescein angiography (FA) followed by systemic perfusion with fluorescently labeled dextran to test vascular permeability of both albumin-bound (66 kDa) fluorescein and 10 kDa dextran. In PBS-treated *Pf4-DTR* mice, 7 of 9 eyes showed completely normal vascular permeability in both assays (Supplemental Figure 7A). In 2 of 9 eyes, vascular permeability was increased predominantly in the 10 kDa dextran assay with marginal FA findings (Supplemental Figure 7B). In DT-treated *Pf4-DTR* mice, 6 of 9 eyes were normal (Supplemental Figure 7C), whereas 3 of 9 eyes showed increased vascular permeability (Supplemental Figure 7D). There was no significant difference between PBS-treated eyes (22%) and DT-treated eyes (33%) (*P* = 0.28).

Next, we tested the role of perivascular macrophages in immune cell infiltration by repeating our PBS and DT protocol followed by intravitreal CCL2 injections on day 4 and sacrificed *Pf4-DTR* mice on day 5. DT-mediated ablation of perivascular macrophages decreased CCL2-driven Ly6C^+^ cell infiltration by 75.8% (*P* < 0.01, Figure 7, D–H). These data suggest that perivascular macrophages were necessary for acute CCL2-driven neuroinflammation.

Finally, we repeated the above study and additionally performed both FA and 10 kDa dextran perfusion assays. FA showed vascular hyperpermeability around the optic nerve head in PBS- and DT-treated eyes that received intravitreal CCL2 injections (Supplemental Figure 8, A and B). The number of Ly6C^+^ cells was markedly reduced in DT-treated eyes, while the 10 kDa dextran leakage was reduced to similar or lesser degrees (Supplemental Figure 8, C and D). Optical coherence tomography (OCT) showed dramatically reduced immune cell extravasation near the optic nerve (Supplemental Figure 8, E and F). Total vascular leakage showed a trend toward a reduction in DT-treated eyes (Supplemental Figure 8G). Since immune cell infiltration will secondarily affect vascular permeability, this underpowered study suggests that perivascular macrophage ablation primarily affects immune cell extravasation, but further investigation is warranted.

## Discussion

In this report, we comprehensively characterized perivascular macrophages in the retina to determine their function. We found that retinal perivascular macrophages reside on 20–40 μm postcapillary venules (Figures 2 and 3), are targetable with *Pf4^Cre^* mice (Figure 4), and express a prochemotactic transcriptome (Figure 5). On the basis of these data, we hypothesized that perivascular macrophages are necessary for immune cell infiltration during acute neuroinflammation. This hypothesis is supported by prior reports that immune cell transendothelial migration occurs at postcapillary venules in multiple organ systems (12) including the retina (25), that macrophage–endothelial cell crosstalk can orchestrate immune cell infiltration (11), and that *Pf4*/CXCL4 regulates immune cell recruitment (26). We modeled neuroinflammation with a single CCL2 intravitreal injection and found that perivascular macrophages were associated with Ly6C^+^ cells (Figure 6) and that perivascular macrophage depletion significantly reduced Ly6C^+^ cell recruitment (Figure 7). These data suggest that perivascular macrophages are key members of the BRB and orchestrate immune cell recruitment during neuroinflammation.

Although retinal perivascular macrophages are extremely understudied, perivascular macrophages have been investigated in other neural tissues. In the brain, perivascular macrophages are similarly CD206^+^ and reside within the vascular sheath (3). Their functions include the regulation of immune cell chemotaxis and vascular permeability (3, 10, 27), in partial agreement with our studies. We also found that retinal perivascular macrophages regulated immune cell infiltration (Figure 7). However, there was no significant effect of retinal perivascular macrophage ablation at steady state on vascular permeability (Supplemental Figure 7). After CCL2 intravitreal injection, perivascular macrophage ablation showed a trend toward less vascular permeability, but the effect size was equal or less than immune cell chemotaxis. Since immune cell infiltration can secondarily affect vascular permeability, our data support a more substantial effect of perivascular macrophages on chemotaxis than on permeability. This conclusion is supported by our scRNA-Seq data, which showed increased expression of chemotaxis genes but no change in the expression of extracellular matrix or permeability genes. Nevertheless, these studies are underpowered and warrant further investigation.

Additionally, brain perivascular macrophages express MHCII and are capable of antigen presentation (28, 29), also in agreement with our findings. In partial alignment with our findings, brain perivascular macrophages reside on venules and arterioles, but not capillaries (9). We replicated these data in the brain and found no arteriolar or venular preference (Supplemental Figure 4), but a clear preference for venules in the eye (Figure 3). These interesting differences may be related to vessel size and warrant further investigation. Furthermore, perivascular macrophages in the brain are derived from meningeal macrophages and demonstrate heterogeneity (9, 30), two important areas of future investigation in the retina. We similarly found heterogeneity in the expression of MHCII in retinal perivascular macrophages. In the spinal cord, perivascular macrophages also express MHCII heterogeneously and regulate vascular permeability through extracellular matrix protein levels in a model of amyotrophic lateral sclerosis (31). Finally, both brain and dorsal root ganglion perivascular macrophages express CD163 (28, 32). Like in the retina, CD163^+^ dorsal root ganglion perivascular macrophages differentially express *Pf4* and *Ccl24* and, like in the brain, regulate endothelial permeability (32). These data demonstrate potential conserved functions across tissues between CNS perivascular macrophages in terms of antigen presentation, vascular permeability, and immune cell chemotaxis.

Since retinal perivascular macrophages are necessary for CCL2-driven immune cell infiltration, they may play an important role in the pathophysiology of DR. In murine DR models, retinal *Ccl2* expression is upregulated; furthermore, *Ccl2^–/–^* and *Ccr2^–/–^* mice both show reduced immune cell leukostasis and infiltration, decreased vascular permeability, and less endothelial cell death (33, 34). These data demonstrate the pathogenic role of classical monocytes and classical monocyte–derived macrophages in DR progression and breakdown of the BRB during murine DR. In both mice and humans, BRB disruption is associated with a more advanced DR stage (1), and CCL2 levels are increased in patients with DR and correlate with diabetic macular edema (23), a marked cause of vision loss in patients with DR. Furthermore, intravitreal steroids are an effective treatment for both diabetic macular edema and regression of DR stage in clinical trials (35, 36), demonstrating the importance of inflammation and BRB breakdown in human DR. Finally, imaging of macrophage-like cells at the vitreoretinal interface in patients with DR shows that the number of these cells increases with advanced DR stage, diabetic macular edema, and ischemia (37–41). Macrophage-like cells at the vitreoretinal interface have the potential to include microglia, perivascular macrophages, vitreal hyalocytes, and recruited inflammatory cells (13). Together, these data suggest that BRB breakdown leads to inflammatory cell recruitment, resulting in diabetic macular edema and DR stage progression, which includes proliferative DR (PDR) with neovascularization. In agreement with this, scRNA-Seq of human PDR membranes demonstrates that nonmicroglial macrophages are present and express high amounts of *VEGFA* (42). Thus, perivascular macrophages are potential key pathogenic cells in both BRB breakdown and immune cell infiltration, which are central processes in the pathophysiology of DR.

Retinal perivascular macrophages may also play important roles in the pathophysiology of noninfectious intraocular inflammation or uveitis. Similar to DR, retina-involving uveitis is a disease that includes breakdown of the BRB. In mice, uveitis is modeled using the experimental autoimmune uveitis (EAU) model. In EAU, mice are immunized with interphotoreceptor retinoid–binding protein (IRBP). IRBP is expressed by photoreceptor outer segments, and immunization targets the immune response to photoreceptors (43). For initiation of EAU to occur, both MHCII-dependent antigen presentation and retinal macrophages are necessary (43, 44). In addition, early immune cell infiltration during EAU occurs from large vessels in the superficial vascular plexus (45). Furthermore, the early events of EAU include leukocyte adhesion to postcapillary venules, breakdown of the BRB, and, finally, immune cell infiltration into the retinal parenchyma (46). Since perivascular macrophages are the predominant MHCII^+^ retinal macrophages, are found in the superficial vascular plexus, and are predominantly associated with venules, future investigations into the role of perivascular macrophages in EUA are warranted.

This study has several limitations. First, our *Pf4^Cre^* model also targets hyalocytes. However, DT-driven ablation did not reduce hyalocyte levels. This is likely either because hyalocytes exist within the BRB barrier or because of the high density of hyalocytes and perivascular macrophages that exist over the optic nerve head. This area was too dense to definitively discriminate between hyalocytes and perivascular macrophages with high certainty. Thus, we cannot exclude the possibility that our reduction in immune cell chemotaxis after CCL2 intravitreal injections was not due to hyalocyte depletion. Second, we did not investigate the effects of perivascular macrophage depletion on endothelial cells. Since perivascular macrophages are only present on large venules in the eye, a bulk RNA-Seq approach of sorted endothelial cells would likely not yield marked differences due to the many capillary endothelial cells without perivascular macrophage contact. To determine how perivascular macrophages influence endothelial cells, a scRNA-Seq or spatial transcriptomics approach would be necessary to understand how large postcapillary venule endothelial cells change after perivascular macrophage ablation, which is beyond the scope of this current manuscript. Third, we also did not study how perivascular macrophage ablation affects extracellular matrix proteins like collagen IV and laminin, which have been shown to be regulated by perivascular macrophages in amyotrophic lateral sclerosis (31). In the Adachi et al. study (31), these extracellular matrix proteins were affected by perivascular macrophage ablation that occurred over 6 weeks. Therefore, more chronic ablation is likely necessary to uncover these changes and is an area of future investigation. Finally, *Pf4*/CXCL4 is a chemokine expressed in other cells external to the retina. Although *Pf4* was not expressed in extravasating Ly6C^+^ monocytes (Figure 6H), we cannot exclude an effect of reduced CXCL4 expression in stromal or nonmonocyte hematopoietic bone marrow or blood cells prior to ocular infiltration.

In summary, retinal perivascular macrophages express MHCII, are located on postcapillary venules, and are targetable with *Pf4^Cre^* mice. Furthermore, *Pf4^+^* macrophages express a prochemotactic transcriptome, correlate with immune cell chemotaxis, and are necessary for Ly6C^+^ immune cell infiltration after intravitreal CCL2 injection. These data suggest that perivascular macrophages are key members of the BRB that regulate immune cell transendothelial migration and may play important roles during DR and uveitis pathophysiology.

## Methods

### Sex as a biological variable.

All studies were carried out on male and female mouse populations, with a minimum of 2 animals per sex per group. All data were investigated for sex-specific effects and none were found.

### Animals.

WT C57BL/6J (no. 000664), *Tmem119^GFP^* (no. 031823), *Rosa26^CAG-LSL-ZsGreen1^* (no. 007906), *Rosa26^CAG-LSL-DTR^* (no. 007900), and *Pf4^Cre^* (no. 008535) mice were purchased from The Jackson Laboratory. WT C57BL/6J were bred and maintained within a pathogen-free barrier environment at the Center for Comparative Medicine at Northwestern University. *Tmem119^GFP^* mice were crossed with WT mice to generate *Tmem119^GFP/+^* mice for experiments. *Pf4^Cre^* mice were bred with *Rosa26^CAG-LSL-ZsGreen1^* and *Rosa26^CAG-LSL-DTR^* mice to generate *Pf4^Cre^:Rosa26^zsGreen/+^* (*Pf4*-zsGreen) and *Pf4^Cre^:sa26^CAG-LSL-DTR/+^* (*Pf4-DTR*) mice for experiments, respectively. Genotyping was performed by Transnetyx to confirm the absence of the RD8 allele (*Crb1^–^*). Unless otherwise specified, all experiments were carried out using 10- to 12-week-old mice.

### Flow cytometry of mouse retina.

Following enucleation, eyes were transported in HBSS (14025076, Gibco, Thermo Fisher Scientific) and dissected under a microscope to isolate retina. Retina-only samples were digested and processed identically to our previously published flow cytometry procedure, with the exception that no mechanical digestion was performed (47). A single-cell suspension was prepared and then stained with immune cell markers (Supplemental Table 1). All experiments were analyzed on a FACSymphony A5-Laser Analyzer (BD), and data were analyzed with FlowJo version 10 software.

### Immunofluorescence imaging of retinal flatmounts.

After mice were sacrificed, eyes were enucleated and fixed in 4% paraformaldehyde (15713-S; Electron Microscopy Sciences) for 1 hour at room temperature. Retinas were isolated in 1× Tris-buffered saline (TBS) and stored in TBS plus 5% donkey serum (S30, MilliporeSigma) plus 2.5% BSA (A2153, MilliporeSigma) plus 0.5% Triton X-100 (X100, MilliporeSigma) overnight at 4°C. Primary incubations were performed overnight at 4°C (Supplemental Table 1). Next, retinas were washed with TBS-T (TBS with 0.5% Tween-20; 00777, Amresco) 5 times and incubated with secondary antibodies overnight at 4°C (Supplemental Table 1). Retinas were then washed with TBS-T and mounted on HistoBond microscope slides (16004–406, VWR) with Immu-Mount (9990402, Thermo Fisher Scientific). Imaging was performed on a Nikon W1 Dual CAM Spinning Disk Microscope using Nikon NIS Elements software. The approximate distances between vascular plexuses were 8–11 μm between superficial and intermediate and 8–12 μm between intermediate and deep.

### Immunofluorescence imaging of frozen sections.

Eyes were processed identically to our previously published work (13). For frozen brain sections, mice were perfused with ice-cold HBSS, and brains were extracted from the skull and cut along the median longitudinal fissure. Brains were fixed in 4% paraformaldehyde at room temperature for 2 hours. Brains were washed in PBS with10%, 20%, and 30% sucrose, identically to eyes. Next, the brains were placed in a 25 mm × 20 mm × 5 mm vinyl specimen mold (4557, Sakura) with the sliced side on the bottom of the mold and embedded in optical cutting temperature compound (23-730-571, Fisher Healthcare). The samples were frozen at –80°C and cut into 8 μm sections with a cryostat. Eye and brain sections were stained identically. Slides were washed in PBS for 10 minutes, blocked for 1 hour at room temperature in 5% donkey serum, and then stained identically to retinal flatmounts. Immunofluorescence was performed on a Nikon Ti2 Widefield using Nikon NIS Elements software.

### MotiQ analysis.

Fifty microglia, 25 perivascular macrophages, and 25 hyalocytes were selected from the superficial vascular plexus (*n* = 5–6 mice) for MotiQ analysis, as previously described (16). The total area, spanned area, tree length, and ramification index were determined.

### scRNA-Seq analysis.

scRNA-Seq data previously published by O’Koren et al. were analyzed using Spectacle (singlecell-eye.org) (17, 18). Our own prior scRNA-Seq data were analyzed using Seurat version 4 (19, 20, 48). This analysis used only RNA-Seq data from control no-laser eyes or control no-laser choroid/retina from WT, *Ccr2^–/–^*, and *Nr4a1^–/–^* mice. Quality control metrics were identical to those in our prior reports (19, 20). Data were independently normalized, scaled, and then integrated using reciprocal principal component analysis (49). Clustering was performed using the standard workflow with 22 principal components and 0.4 resolution. A mononuclear phagocyte subset was created, rescaled, renormalized, and reclustered using 17 principal components and 0.7 resolution. The Wilcoxon rank-sum test was used for differential expression using the FindAllMarkers function within Seurat (>10% of cells, log_2_fold change [FC] >0.59 [1.5-fold] between the cluster of interest and all other cells). We chose the 1.5-fold cutoff so that were enough differentially expressed genes in each cluster for GO enrichment analysis. GO enrichment analysis was performed on genes upregulated more than 1.5-fold, with an adjusted *P* value of less than 0.01. GO enrichment was performed using Gorilla (50), with a background of genes expressed in greater than 5% of mononuclear phagocytes. Selected GO terms are shown in Figure 5E; all GO terms are listed in Supplemental Table 3. The DotPlot function was used to visualize genes that were included in selected GO terms.

### Intravitreal injections.

Mice were anesthetized, eyes were dilated, and pain prophylaxis was provided as previously described (51). Intravitreal injections were performed identically to our previously report using 1 ng CCL2 in 1 μL sterile PBS (13). Retinal flatmount immunofluorescence imaging or flow cytometry was performed 18 hours after intravitreal injections.

### DT administration.

Intraperitoneal injections of sterile PBS vehicle control or 200 ng diphtheria toxin (322326-1MG, MilliporeSigma) in 0.1 mL PBS were administered for 4 consecutive days. Intravitreal injections were given on day 4. Mice were sacrificed on day 5 for immunofluorescence of retinal flatmounts or flow cytometry.

### Optical coherence tomography and vascular permeability assays.

Mice were anesthetized, eyes were dilated, and pain prophylaxis was provided as previously described (51). Mice were given a single intraperitoneal injection of 0.05 mL AK-FLUOR 25% (sodium fluorescein, 250 mg/mL) for FA. Mice were next positioned on the Spectralis OCT2 system (Heidelberg Engineering) for FA and OCT imaging. A contact lens was placed on the surface of the eye (Cantor and Nissel, 3.2 mm diameter, 1.7 mm base curve, no. 90,642). Late-phase FA images were taken 5 minutes after fluorescein injection with 100-frame averaging for high resolution. High-resolution OCT images at the optic nerve were taken, with an average of 100 frames. Mice were next given a retro-orbital injection of 10 kDa Dextran (Invitrogen, Thermo Fisher Scientific, D22914, 5 mg/mL), as previously described (52). Five minutes after retro-orbital injection, eyes were processed for confocal microscopy as described above. Total leakage was calculated in Fiji using the ROI manager function to delineate the leakage area and measure both area and mean intensity. The area was multiplied by mean intensity for total leakage per eye.

### Statistics.

For the flow cytometric studies, CD206 MFI, CD169 MFI, and MHCII MFI and cell counts were compared using repeated-measures, 1-way ANOVA followed by Tukey’s multiple-comparison test. For immunofluorescence studies, macrophage subset counts were compared using repeated-measures, 1-way ANOVA followed by Tukey’s multiple-comparison test or a paired, 2-tailed *t* test. For MotiQ analysis, 1-way ANOVA followed by Tukey’s multiple-comparisons test was performed. Overlaps between perivascular macrophages and Ly6C^+^ cells were compared using a transformation of the Kolmogorov-Smirnov test. The percentage of depletion of cells was compared using 2-way ANOVA followed by Šidák’s multiple-comparison test. Ly6C^+^ monocyte density was compared using the Mann-Whitney *U* test because the distribution was nonparametric on the Shapiro-Wilk test. A *P* value of less than 0.05 was considered statistically significant. All data are presented as the mean ± SEM.

### Study approval.

All studies adhered to the Association for Research in Vision and Ophthalmology (ARVO) Statement for Animal Use in Ophthalmic and Vision Research and received approval from the IACUC of Northwestern University.

### Data availability.

Raw scRNA-Seq data are available in the Gene Expression Omnibus (GEO) database (GEO GSE239941 and GSE222094). All other data, including specific code, are available upon reasonable request to the corresponding author. All data values in graphs are available in the Supporting Data Values (raw graph data) file.

## Author contributions

JKS’s roles included conceptualization, formal analysis, data curation, investigation, methodology, visualization, and writing of the original draft. AR’s role was investigation, formal analysis, methodology, and writing, reviewing, and editing of the manuscript. SD’s roles included data curation, investigation, methodology, and writing, reviewing, and editing of the manuscript. JG’s roles included investigation and writing, reviewing, and editing of the manuscript. ALW’s roles included data curation, investigation, and writing, reviewing, and editing of the manuscript. APV’s roles included data curation, formal analysis, and writing, reviewing, and editing of the manuscript. CEB’s roles included investigation and writing, reviewing, and editing of the manuscript. JAL’s roles were conceptualization, data curation, formal analysis, funding acquisition, investigation, methodology, project administration, supervision, visualization, and writing the original draft as well as editing and reviewing of the manuscript. JKS was chosen as the first author, despite equal overall contribution with AR, due to his role in conceptualization and writing of the original draft.

## Supplementary Material

Supplemental data

Supplemental table 2

Supplemental table 3

Supporting data values

## Version 1 08/29/2024

In-Press Preview

## Version 2 10/15/2024

Electronic publication
