# Supplementary material for: Retinal perivascular macrophages regulate immune cell infiltration during neuroinflammation in mouse models of ocular disease
Source: J Clin Invest. 2024 Aug 29;134(20):e180904. doi: 10.1172/JCI180904 (PMC11473146; doi:10.1172/JCI180904)
Supplement: Supplemental data [file jci-134-180904-s049.pdf]

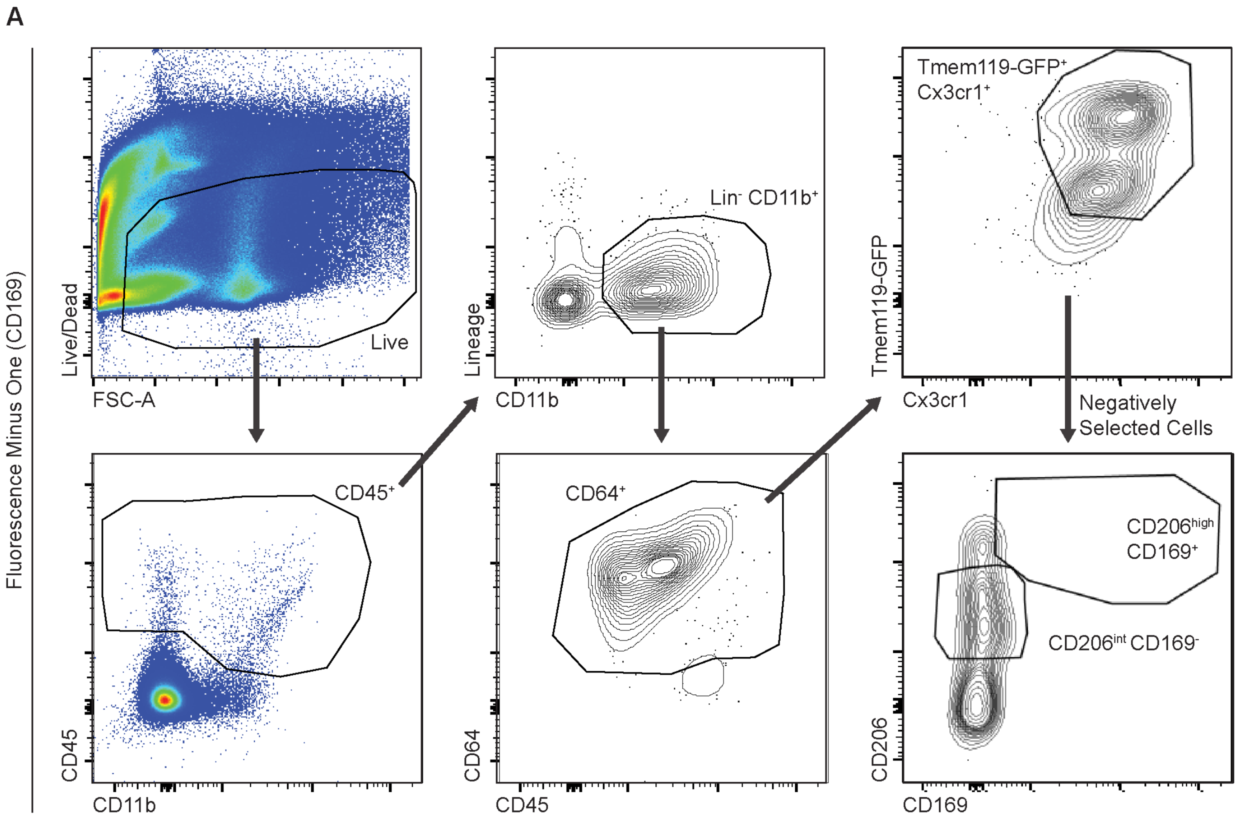

Supplemental Figure 1: Fluorescence minus one control from CD169 multi-parameter flow cytometry studies. See Figure 1 for gating strategy details and Figure 1A (lower right panel) for direct comparison.

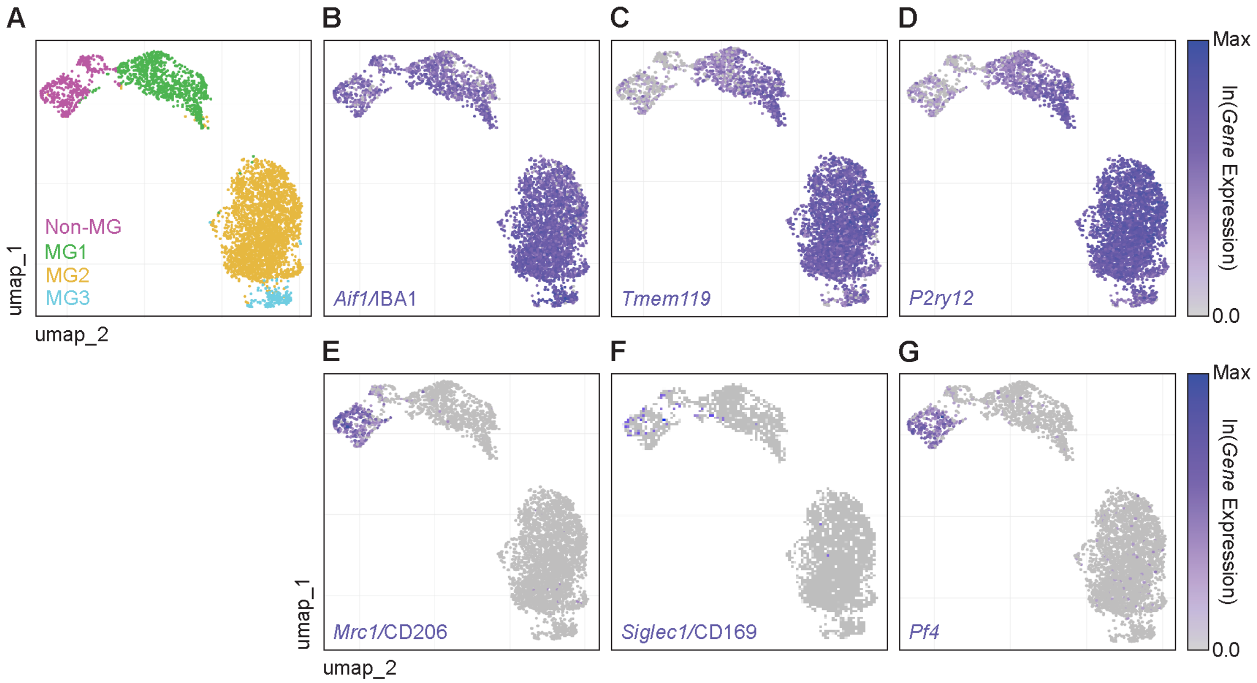

Supplemental Figure 2: Non-microglia express greater *Pf4* and *Mrc1* than microglia. A: UMAP dimension plot. B-G: Feature plots for *Aif1*/IBA1, *Tmem119*, *P2ry12*, *Mrc1*/CD206, *Siglec1*/CD169, and *Pf4*/CXCL4.

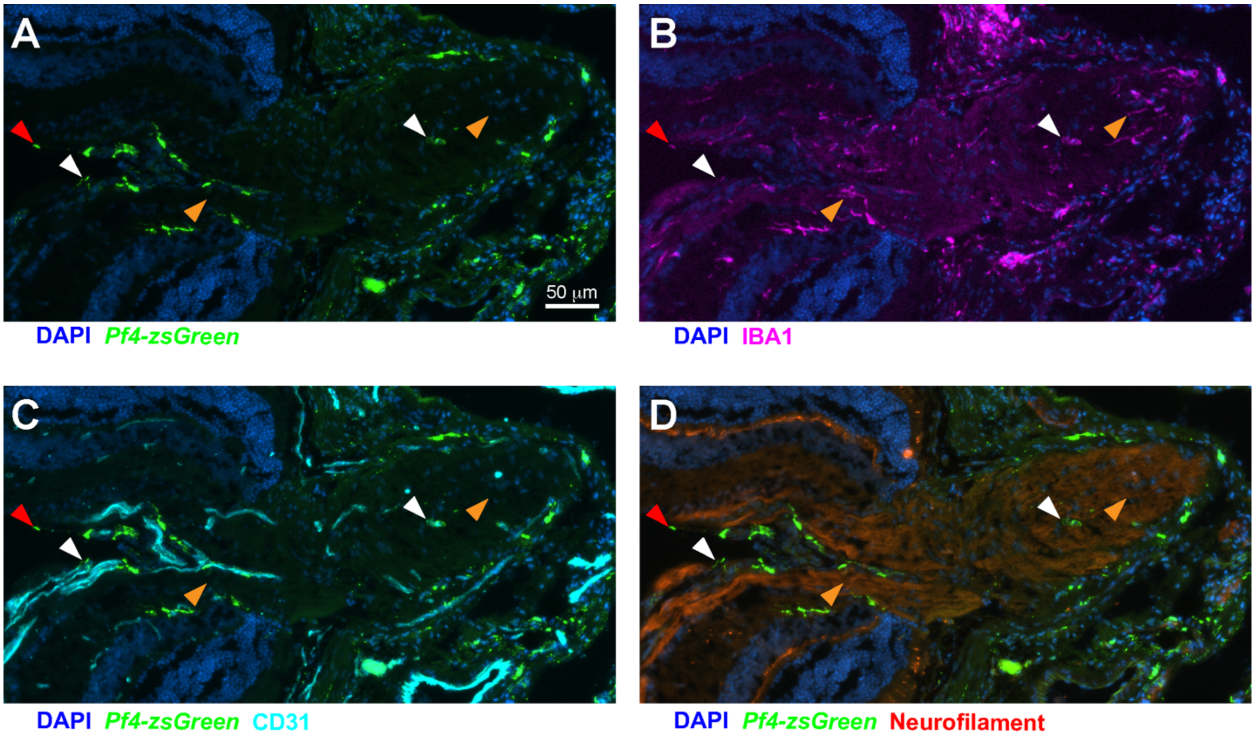

Supplemental Figure 3: Optic nerve head macrophages include both perivascular macrophages and hyalocytes. Representative immunofluorescence section through the optic nerve head from *Pf4-zsGreen* mice. Arrowheads indicate hyalocytes (red), perivascular macrophages (white), and microglia (orange).

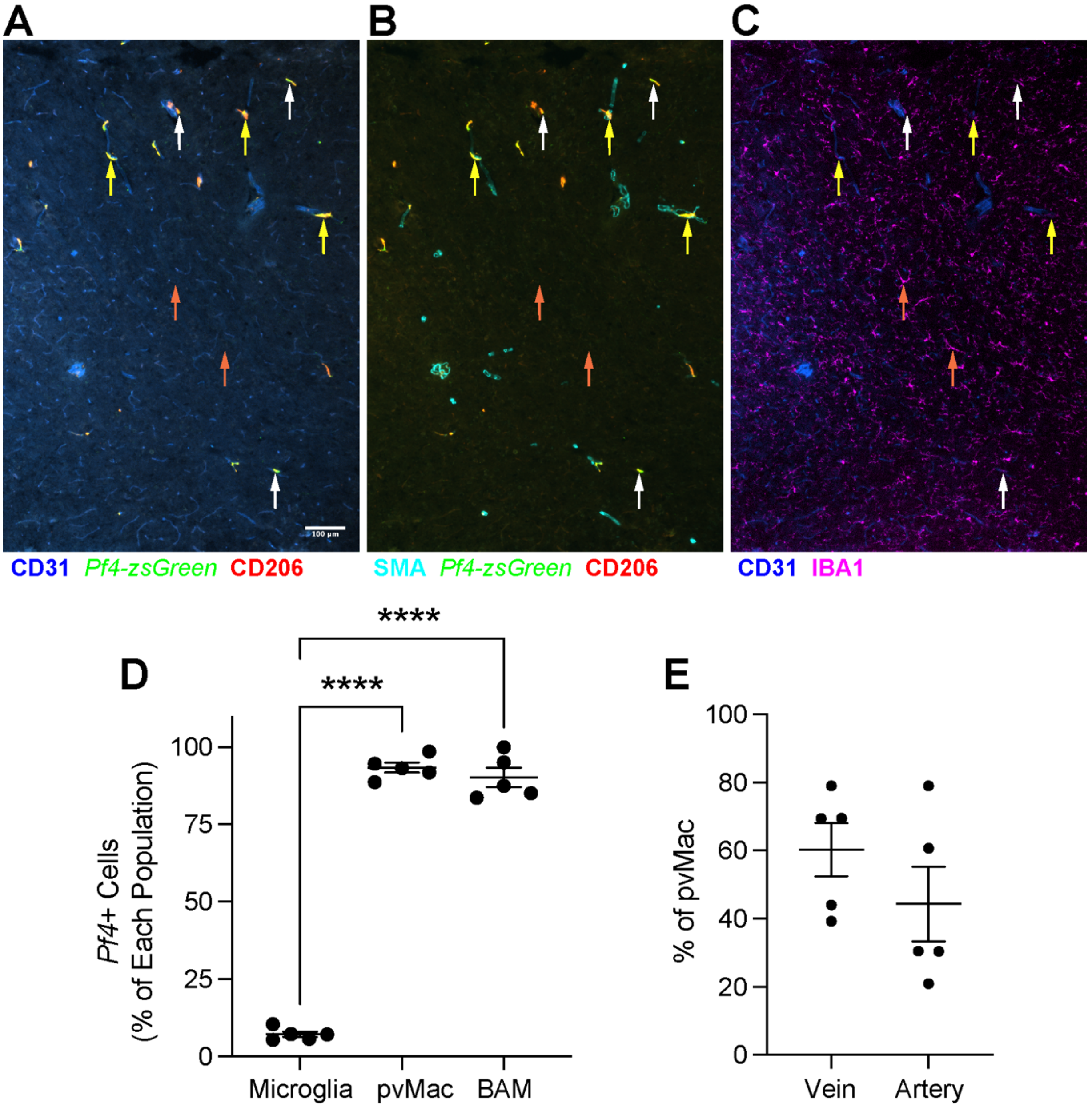

Supplemental Figure 4: Brain perivascular macrophages are *Pf4*+ but present in arteries and veins equally. A-C: Representative frozen section of the brain from *Pf4-DTR* mice. Yellow arrows highlight perivascular macropahges (pvMac) on arterioles, white arrows show pvMac on venules, and orange arrows demonstrate microglia. D: Perivascular macrophages (pvMac) and border-associated macrophages (BAMs) are nearly 100% *Pf4*$^{+}$. E: Perivascular macrophages (pvMac) are equally found on arterioles and venules in brains. Data were analyzed by repeated measures ANOVA followed by Tukey's multiple comparisons test: **** $p<0.0001$ (D) or paired t-test (E, not significant).

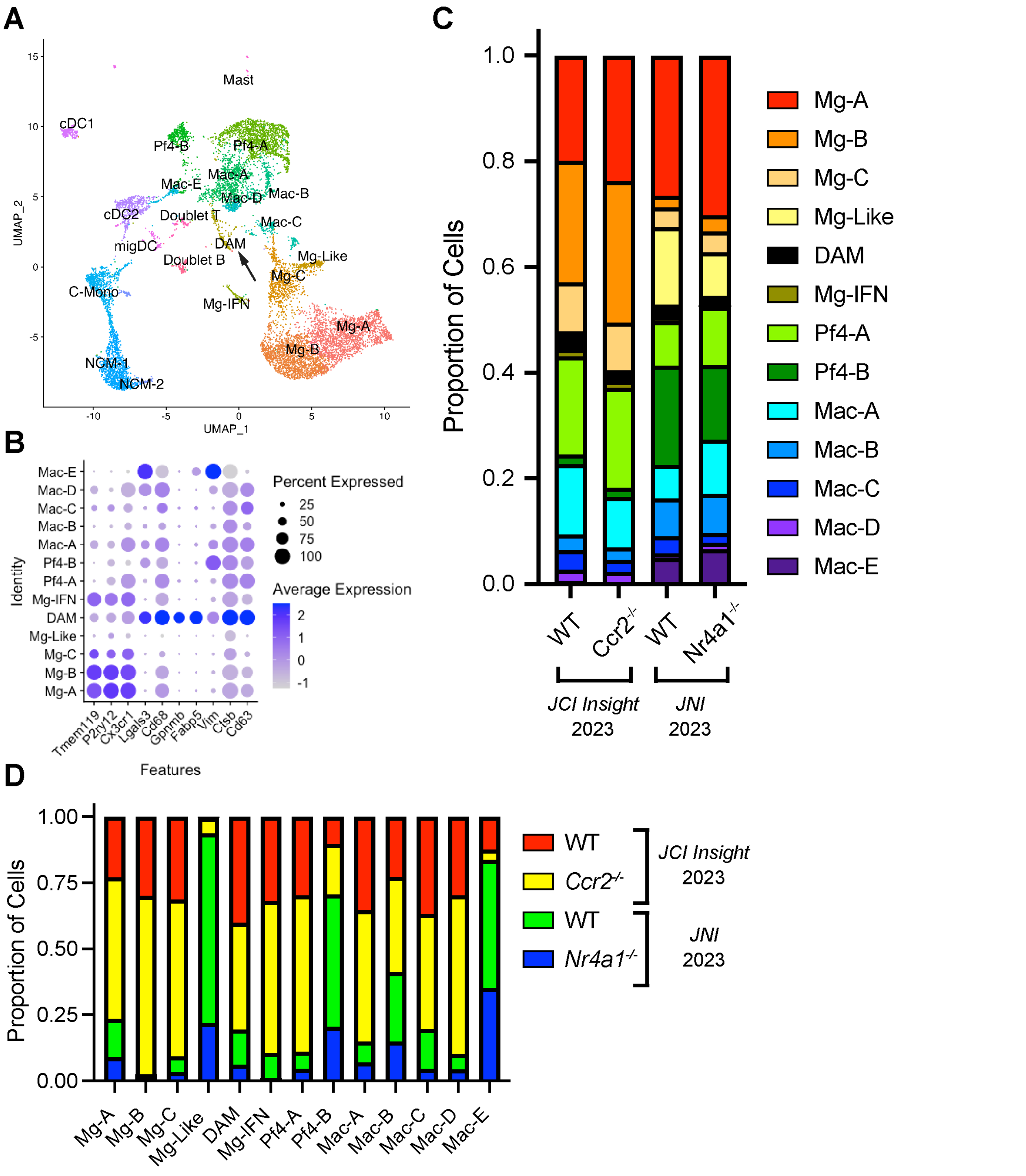

Supplemental Figure 5. Disease-associated microglia (DAM) likely correspond to previously published sub-retinal microglia. A: UMAP dimension plot from Fig 5A with arrow identifying DAM cluster. B: DotPlot demonstrating reduced microglia homeostasis genes (Tmem119, Pr2ry12, Cx3cr1) and increased sub-retinal microglia genes (Lgals3 – Cd63) in the DAM cluster. C: Proportion of cells from each sample showing that DAMs (black) represent 1-3% of total macrophages. D: Proportion of cells from each cluster.

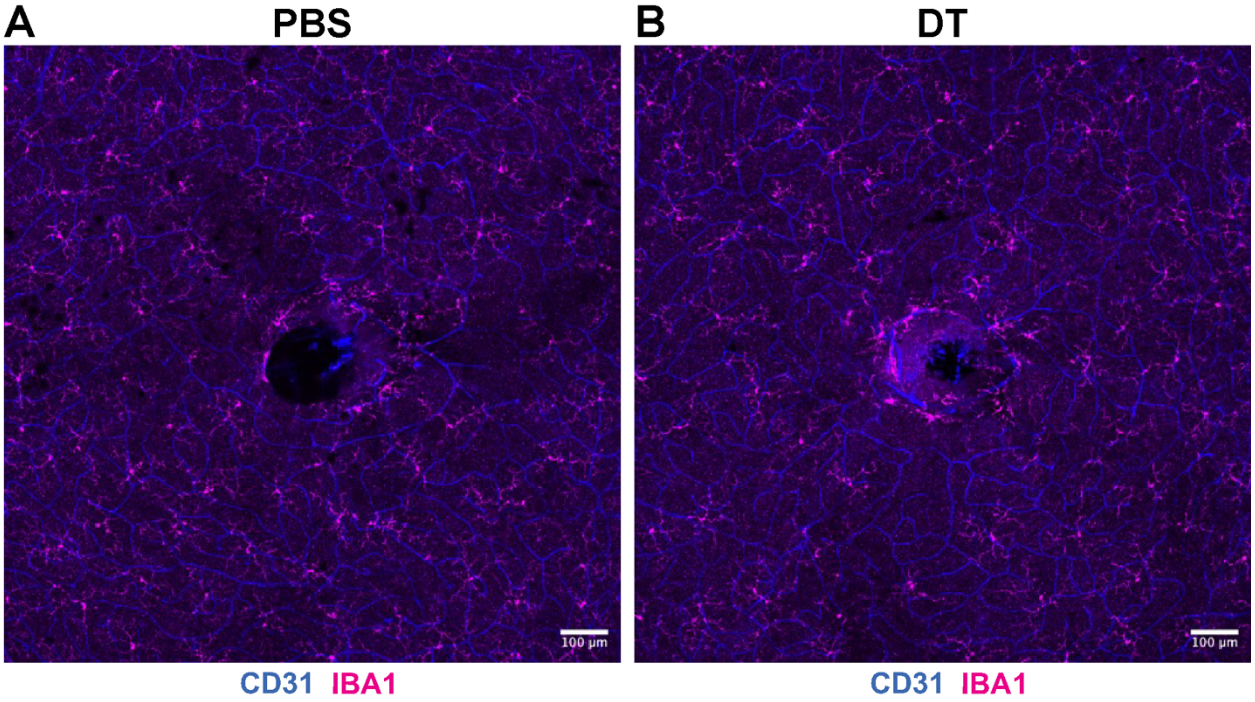

Supplemental Figure 6: Microglia are unaffected by DT treatment in *Pf4-DTR* mice. IBA1 macrophages are unchanged in the deep vascular plexus after DT treatment.

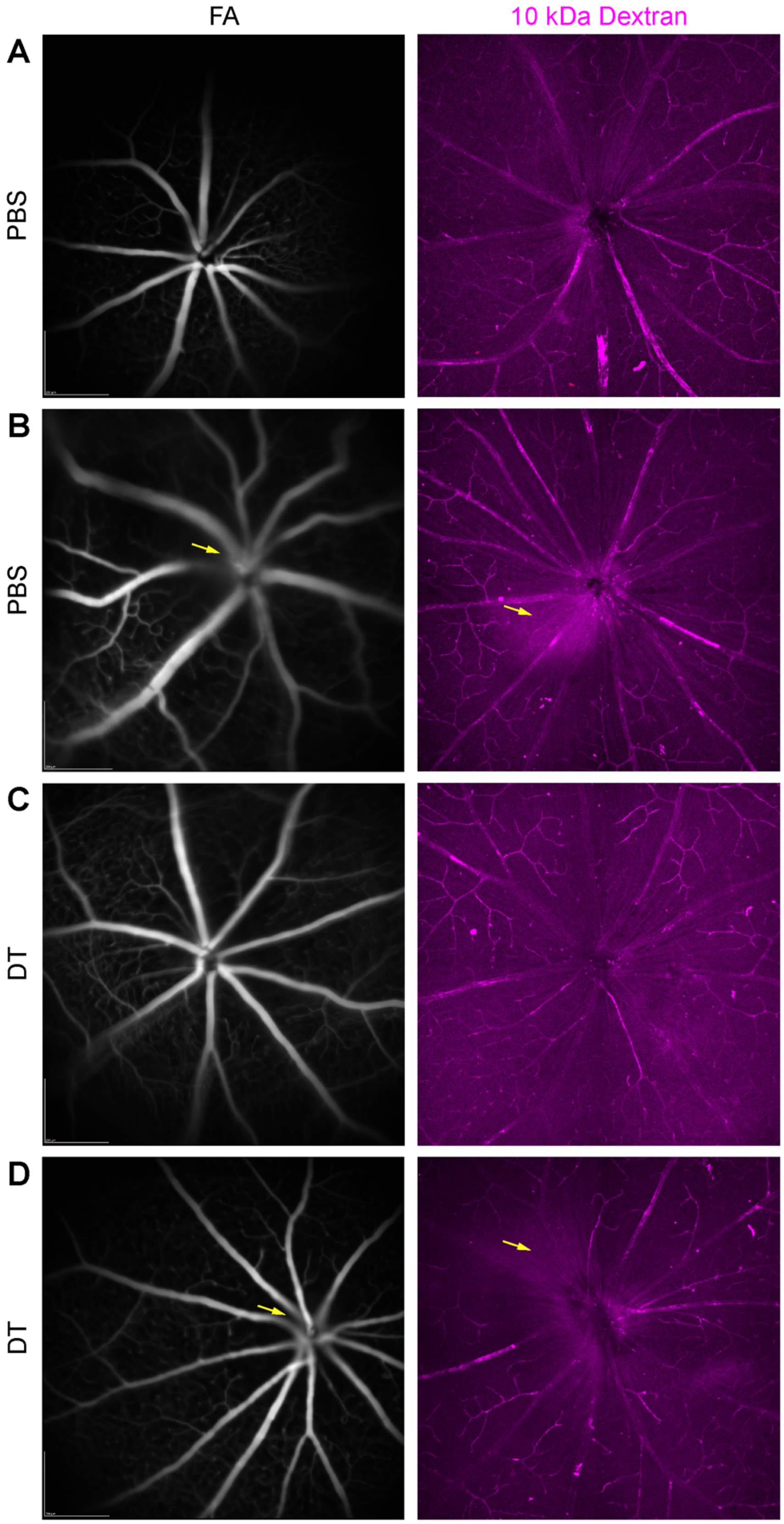

Supplemental Figure 7. *Pf4-DTR* mice display no change in vascular permeability at steady-state. A-B: Fluorescein angiography (FA) and 10 kDa Dextran permeability assays in PBS-treated *Pf4-DTR* mice. Seven of 9 eyes showed normal vascular permeability, and 2 of 9 eyes showed increased permeability near the optic nerve head (yellow arrows). C-D: Fluorescein angiography and 10 kDa Dextran permeability assays in DT-treated *Pf4-DTR* mice. Six of 9 eyes showed normal vascular permeability, and 3 of 9 eyes showed increased permeability near the optic nerve head (yellow arrows).

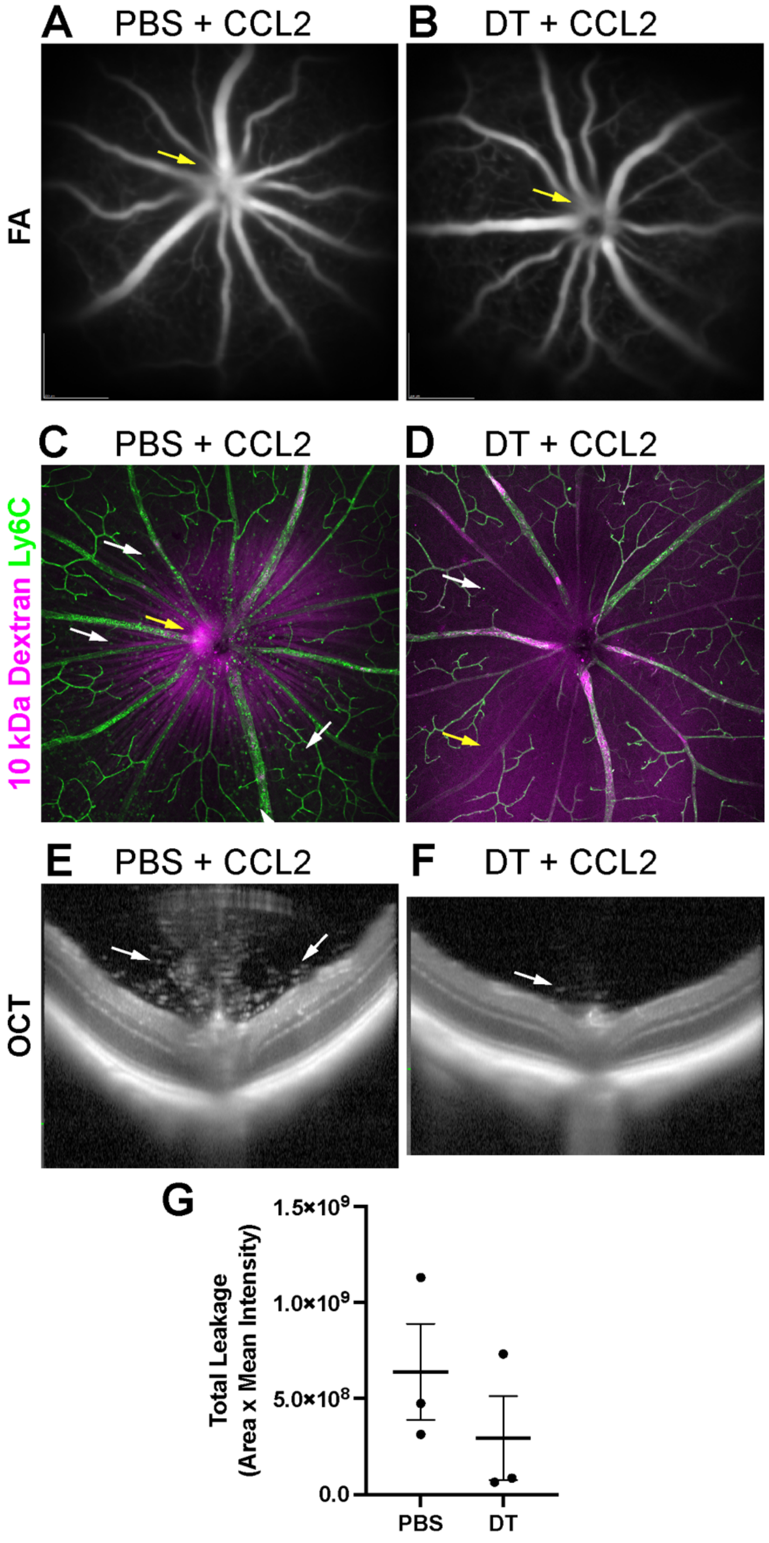

Supplemental Figure 8. *Pf4-DTR* mice display mild changes in vascular permeability after CCL2 injection. A-B: Fluorescein angiography (A-B) from PBS (A) and DT (B) treated *Pf4-DTR* mice 18 hours after CCL2 intravitreal injection. Yellow arrows indicate mildly increased permeability by the optic nerve head. C-D: Ly6C and 10 kDa dextran staining from eyes in A-B. White arrows indicate Ly6C$^+$ cells while yellow arrows indicate increased vascular permeability. DT-treated mice show mildly reduced permeability. E-F: Optical coherence tomography (OCT) images through the optic nerve head show severely diminished inflammatory cells after CCL2 injection in DT-treated mice. G: Total vascular leakage was mildly reduced in DT-treated eyes but similar to the reduction in inflammatory cells.

Supplemental Table 1. Antibodies used for flow cytometry and immunofluorescence studies.

Antibody	Fluorophore	Dilution	Manufacturer	Use
Goat anti-mouse CD31	-	1:250	R&D Systems, AF3628	IF
Rabbit anti-mouse IBA1	-	1:500	Wako, 019–19,741	IF
Chicken anti-mouse GFP	-	1:6000	Abcam, ab13970	IF
Chicken anti-mouse Neurofilament	-	1:1000	Abcam, ab134458	IF
Lectin (biotinylated)	-	1:200	Vector Laboratories B-1205	IF
Smooth Muscle Actin	-	1:500	Invitrogen, MA1-06110	IF
Goat anti-human DTR (Human HB-EGF)	-	1:250	R&D Systems, AF259	IF
Rat anti-mouse Ly6C (biotinylated)	-	1:1000	Abcam, ab54223	IF
Donkey anti-goat (H + L)	Alexa Fluor 405	1:500	Invitrogen, A48259	IF
Donkey anti-chicken (H + L)	Alexa Fluor 488	1:500	Jackson Immuno, 703-545-155	IF
Donkey anti-goat (H+L)	Alexa Fluor 488	1:500	Invitrogen, A11055	IF
Donkey anti-rat (H + L)	Alexa Fluor 555	1:500	Invitrogen, A78945	IF
Streptavidin	Alexa Fluor 555	1:500	Invitrogen, S32355	IF
Donkey anti-rabbit (H + L)	Alexa Fluor 647	1:500	Invitrogen, A31573	IF
Fc Block			BD Biosciences, 553,142	Flow
Aqua Live/Dead	AmCyan		ThermoFisher, 65–0866-14	Flow
CD45	BUV395		BD Biosciences, 564279	Flow
CD64	BV786		BD Biosciences, 741024	Flow
CD11b	APC-Cy7		BD Biosciences, 557657	Flow
Cx3Cr1	Alexa Fluor 647		Biolegend, 149033	Flow
CD169	PE		Biolegend, 142404	Flow
CD206	PE-Cy7		Biolegend, 141720	Flow
Ly6G	PE-CF594		BD Biosciences, 562700	Flow
B220	PE-CF594		BD Biosciences, 562313	Flow
NK1.1	PE-CF594		BD Biosciences, 562864	Flow
SiglecF	PE-CF594		BD Biosciences, 562757	Flow
CD4	PE-CF594		BD Biosciences, 562314	Flow
CD8	PE-CF594		BD Biosciences, 562315	Flow
Ly6C	FITC		BD Biosciences, 553104	Flow
Ly6G	PE-Cy7		BD Biosciences, 560601	Flow
MHCII	A700		Biolegend, 107622	Flow

GFP = green fluorescent protein, DTR = diphtheria toxin receptor, IF = immunofluorescence, Flow = flow cytomtery

Supplemental Table Legends

Supplemental Table 2. Differential expression analysis between each macrophage cluster and all other cells. Each tab denotes a cluster of cells including gene names, cluster number, adjusted p-value (p_val_adj), percent of cells expressing gene (pct.1 = current cluster, pct.2 = all other clusters), fold change (avg_logFC = natural log fold change), and raw p-value (p_val).

Supplemental Table 3. Gene ontology (GO) outputs for up-regulated genes from each macrophage subtype. Each tab is from a cluster, includig description of GO term, p-value, FDR q-value, fold enrichment, number of genes expressed in macrophages (N), number of genes in GO term (B), number of differentially expressed genes (n), number of differentially expressed genes in the GO term (b), and the specific genes that were differentially expressed in the GO term. Any cluster not included had no significant GO enrichment.
